# Bank of Standardized Stimuli (BOSS) Phase II: 930 New Normative Photos

**DOI:** 10.1371/journal.pone.0106953

**Published:** 2014-09-11

**Authors:** Mathieu B. Brodeur, Katherine Guérard, Maria Bouras

**Affiliations:** 1 Douglas Mental Health University Institute and Department of Psychiatry, McGill University, Montréal (Québec), Canada; 2 Department of Psychology, Université de Moncton, Moncton (New Brunswick), Canada; 3 Department of Education, University of Sheffield, Sheffield (South Yorkshire), United Kingdom; University of Leicester, United Kingdom

## Abstract

Researchers have only recently started to take advantage of the developments in technology and communication for sharing data and documents. However, the exchange of experimental material has not taken advantage of this progress yet. In order to facilitate access to experimental material, the Bank of Standardized Stimuli (BOSS) project was created as a free standardized set of visual stimuli accessible to all researchers, through a normative database. The BOSS is currently the largest existing photo bank providing norms for more than 15 dimensions (e.g. familiarity, visual complexity, manipulability, etc.), making the BOSS an extremely useful research tool and a mean to homogenize scientific data worldwide. The first phase of the BOSS was completed in 2010, and contained 538 normative photos. The second phase of the BOSS project presented in this article, builds on the previous phase by adding 930 new normative photo stimuli. New categories of concepts were introduced, including animals, building infrastructures, body parts, and vehicles and the number of photos in other categories was increased. All new photos of the BOSS were normalized relative to their name, familiarity, visual complexity, object agreement, viewpoint agreement, and manipulability. The availability of these norms is a precious asset that should be considered for characterizing the stimuli as a function of the requirements of research and for controlling for potential confounding effects.

## Introduction

Stimuli are the key component of experiments. They must therefore be of outstanding quality and be selected meticulously as a function of specific criteria, which explains why they need to be normalized. Normalization is the process through which a representative sample of individuals evaluates images and their names according to specific variables. Normative data characterizes the images and provides a thorough description of their basic features. For instance, by discerning the name given to concepts depicted in images by the majority of individuals, it is possible to determine the level of consensus in naming the specific concepts. The name given by a majority of individuals is the modal name and the consensus is called the name agreement. Through an analysis of the different names given to each image, it is also possible to explain the variability in the given names and to determine how accurately concepts are identified [1]. Other norms commonly tested in sets of pictures include conceptual familiarity, visual complexity, and the typicality of the object. These variables are often normalized because they have a strong influence on many cognitive performances (e.g. object naming) and on the strategies used during image processing.

The need for normative sets of pictures in research is unequivocal and the number of normative studies has rapidly increased in the past years. Indeed, at least 12 new normative sets of pictures, including 2 sets intended to complement older sets [2], [3], were developed between 2000 and 2009 [2]–[13] and 9 new normative sets of pictures were published since 2010 [14]–[22]. Each set is unique about the features of the visual stimuli it includes and the normative dimensions it provides. For example, in Viggiano and colleagues [13]’s dataset, stimuli were normalized in color and in greyscale tones. Op de Beeck and Wagemans [11]’s dataset includes multiple exemplars of each object. Adlington and colleagues [4]’s set includes concepts and images with a broad range of item difficulty and semantic subcategories. Finally, the sets of Barbarotto and colleagues [5] and Magnié and colleagues [10] present imaginary objects, created by combining different objects together. Some sets also offer stimuli normalized for specific visual attributes of the images (e.g. luminosity as opposed to familiarity or visual complexity). For example, the Amsterdam Library of Object Images (ALOI) is a color image set with a large number of images varying in angle, illumination and color. Other sets, such as that of Verfaille and Boutsen [23], use objects in 3-dimensional space instead of line drawings or 2-dimensional images.

The choice between sets of stimuli is made based on each set’s distinctive character and stimulus type. Researchers must first decide whether line drawings or photos of objects are to be used as stimuli. An increasing number of researchers opt for photos of stimuli, highlighting the need for more ecological stimuli. Photos offer a more realistic depiction of everyday concepts. They provide great depth and richness, which potentially influences the way in which the stimulus is attended, memorized and acted upon [24]–[27]. Using photos as the experimental stimuli increases the chances of activating the same neuronal circuits that are activated in daily tasks. Line drawings, such as those created in 1980 by Snodgrass and Vanderwart [28], may also be privileged depending on the researchers’ objective. Line drawings offer a simple and prototypal depiction of concepts, free of details (e.g. color, texture, or 3D cues) that could influence their naming and visual processing. Moreover, line drawings are easier to modify than photos of real objects in order to create additional experimental conditions. They can be made more difficult to recognize by fragmenting their line contours [29] and imaginary and impossible objects can easily be drawn [5], [10].

Once researchers have chosen the type of stimuli they want to use, they have to decide which dimensions they want to control or manipulate in order to determine the set that best suits the needs of the experiment. The number of stimuli available is certainly an important feature that researchers must consider. Experiments often require hundreds of stimuli, especially those including multiple testing sessions, such as memory tasks and experiments involving recording of electrophysiological brain activities. The number of stimuli can be even more crucial for experiments requiring specific types of concepts, such as experiments including specific semantic categories. For example, if the selection of stimuli is limited to the category of fruits and vegetables, only 24 out of the 260 concepts from the Snodgrass and Vanderwart [28]’s set can be used. This issue is usually overcome by combining stimuli from different sets [2]–[3], [30]. However, this practice increases the heterogeneity of the visual parameters and norms.

To our knowledge, the Bank of Standardized Stimuli (BOSS) [15] is the set offering the highest number of normative stimuli (see http://sites.google.com/site/bosstimuli/). It currently includes 538 normative photos of high quality color resolution. In Brodeur and colleagues (2010), stimuli that had a name agreement below 20% or were unrecognized by at least 20% of the participants were excluded from the analyses. Norms presented in this article were thus limited to 480 stimuli. These norms were for the name, familiarity, visual complexity, manipulability, object agreement and viewpoint agreement. Norms are described in more details below. The BOSS, however, does not include some categories that might be useful to researchers, such as animals, vehicles, and buildings. Moreover, a set of 538 images might still be insufficient for some experiments.

The present project further developed the BOSS by adding 930 normative photos. These photos increased the number of stimuli in the existing categories, and offer new categories including animals, building infrastructures, body parts, and vehicles. Differences of norms across categories as well as differences between males and females were also examined. Intrinsic (e.g. biological, neuropsychological, etc.) and extrinsic (e.g. social activities, exposure to specific stimuli, etc.) characteristics of men and women could indeed influence the way they name and rate the concepts. Surprisingly, this has not yet been examined in normative studies.

## Materials and Methods

### Participants

Participants, whose first language is English, were recruited through ads published in journals and newspapers, and via online classifieds such as Craigslist and Kijiji. A total of 141 participants between the ages of 18 and 55 participated in the project. They each participated in one of four normative studies. The subgroups participating in studies 1, 2, 3, and 4 respectively included 42 participants (22 female, mean age: 25.2, SD: 7.5), 33 participants (15 female, mean age: 30.7, SD: 9.3), 32 participants (17 female, mean age: 28.3, SD: 9.9), and 34 participants (15 female, mean age: 30.5, SD: 10.0).

### Ethic Statements

This project was approved by the Research Ethics Board of the Douglas Institute and all participants gave their written consent. Their names were not written anywhere in order to secure confidentiality. Prior to the normative session, participants were told that they were free to interrupt their participation at any time and for any reason. Participants were compensated for their time.

### Stimuli

**Figure 1:**
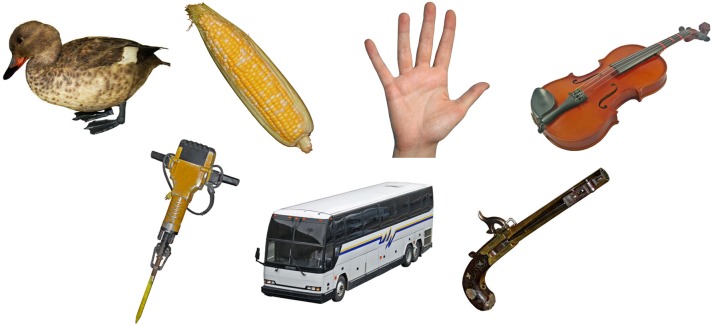
Examples of stimuli from the animal, food, body part, musical instrument, hand labour tool and accessory, vehicle, and weapon and war related item categories.

The 930 new colored photos are all concepts that were not in the original BOSS, except for the cork, ice cube, kiwi, lollipop, mug, and recorder. These concepts were re-normalized by presenting new photos that were considered of better quality than those used in the original BOSS. The new photos depicted concepts of categories that were lacking in the original BOSS, including animals, building infrastructures, body parts, and vehicles. The number of concepts for other categories was significantly increased such as musical instruments, furniture, and weapons. The new set of 930 photo stimuli was created through a 5-step procedure, identical to the procedure used to generate the images for the first phase of the project [15]. Some objects were gathered, cleaned and digitally photographed one at a time in a box that uniformly diffused the light provided by two projectors. Other objects however, were photographed as part of a bigger scene and were then cut out of their backgrounds. These photos were taken in many locations. Consequently, the environmental conditions of the photos were not always uniform. The majority of animal photos were taken in museums and zoos. Few photos were taken from the internet and were generously donated to the project by their authors. Adobe Photoshop (Adobe Systems Inc., San Jose, U.S.A.) was used for image editing, including lighting adjustments and the cutting out of the objects. Examples of photos are presented in Figure 1.

### General Procedure

Stimuli were presented using the software E-Prime 2.0. Participants were tested individually in a room equipped with one desktop computer and one laptop. The desktop was set up with E-Prime and the experiment’s instructions. This computer was used for the stimuli presentation. The photos were presented in 500×500 pixels, centered on the computer screen. On the laptop screen, a blank response sheet was shown in which subjects recorded their responses by writing the name, selecting a category among a list, or entering a value between 1 and 5 on the keyboard. The response sheet was anonymous. The order of the stimuli in each study was random and differed across participants.

#### Study 1

The goal of this study was to normalize the new 930 photo stimuli for name, familiarity and visual complexity. Prior to the experiment, instructions were given orally and a written version was given to each participant. The first task was to “Identify the object as briefly and unambiguously as possible by writing only one name, the first name that comes to mind. The name can be composed of more than one word”. Participants were told to write DKO (don’t know object) if they had no idea what the object was. If they knew the object but not the name, they wrote DKN (don’t know name) and if they knew the name but were unable to retrieve it at that moment, they wrote TOT (tip-of-the-tongue).

For familiarity, participants were asked to “Rate the level to which you are familiar with the object”. Responses were provided on a 5-point rating scale with 1 indicating very unfamiliar and 5 very familiar. Participants were asked to rate the concept itself and not the picture of the object. Responses were not required for the objects for which they responded DKO.

For visual complexity, participants were asked to “Subjectively rate the level to which the image appears to be complex in terms of the quantity of details and the intricacy of the lines”. on a 5-point scale with value 1 indicating a very simple image and 5, a very complex image.

Images were presented one at a time and participants could change to the next image at their own pace, meaning that there was no set amount of time for the participants to see each image. Participants were unable to go back to previous images. For each concept, participants first wrote the name in one column and then provided their rating for familiarity and visual complexity rating in the two next columns of the response sheet. For both familiarity and visual complexity, participants were reminded to use the entire 5-point rating scale and not only its end points.

#### Study 2

The goal of this study was to normalize the photo stimuli for category agreement, which is the extent to which they are representative of their category. In the 2010 normative study (original BOSS) [15], participants classified each object within the most appropriate of 18 categories. This proved problematic when objects fell under more than one category heading. For example, a toy tank could be classified either within the weapon and war related category or within the games, toys and entertainment category. To avoid this problem, the participants in the present study had the possibility to classify the concept within two categories. Considering the change of instructions for this study, the categorization was performed for the 930 new photos as well as for the original 538 normative photos summing to 1468 categorizations.

Categories were created in a drop down box in an excel sheet in alphabetical order. The instructions read, “Determine to which category the concept belongs”. Participants were asked to make a choice among the following five categories: animal, body part, building infrastructure, object, and vehicle. When they chose animal or object, participants were presented with a list of more specific categories allowing them to refine their selection. The list of animals included bird, canine, crustacean, feline, fish, insect, mammal, reptile, and sea mammal. The list of objects included building material, clothing, decoration and gift accessory, electronic device and accessory, food, furniture, game toy and entertainment, hand labour tool and accessory, household article and cleaner, jewel and money, kitchen item and utensil, medical instrument and accessory, musical instrument, natural element, outdoor activity and sport item, skincare and bathroom item, stationary and school supply, weapon and war related item.

#### Study 3

The goal of this study was to normalize the photo stimuli for image agreement, which is the degree to which the mental image generated from the modal name (the name most commonly used), matched the object stimulus. Image agreement was separated into object and viewpoint agreement, meaning that participants had to decide to which extent the mentally generated concept was structurally similar to the photo concept (image agreement) and the extent the two concepts had comparable positions (viewpoint agreement).

For each concept, its name was first presented in black 14-point Times New Roman, centered on the computer screen. This name featured the modal name, which is the name that reached the greatest name agreement, as determined by the results from study 1. Only the 464 stimuli for which at least 21 participants (50%) gave the modal name in study 1 were normalized for object and viewpoint agreement. Following the appearance of the name, participants had to generate a mental image of the concept related to the name, after which, they pressed the space bar and the photo appeared. Participants were then asked to rate image agreement and viewpoint agreement. For object agreement, participants were asked “How closely does the picture of the BOSS resemble the mental image you had for the object name, independently from its position?” For viewpoint agreement, participants were asked to determine “How closely does the object of the BOSS match the position of the object you imagined?” In both tasks, participants had to provide a rating from 1 to 5, 1 corresponding to a low agreement and 5 corresponding to a high agreement. An example of low and high object and viewpoint agreements were presented before the session began.

#### Study 4

In the last study, all 930 stimuli were presented to participants at their own pace in order to rate the manipulability of the concept. Participants were instructed to determine “Could you easily mime the action usually associated with this object so that any person looking at you doing this action could decide which object is associated with this action?” Responses were provided on a 5-point rating scale where 1 was a definite “no” response and 5 was a definite “yes” response. Participants were instructed to use the entire scale and not only its end points.

### Data analyses

#### Modal name and name agreement

For each image, the names provided by participants were analyzed after first excluding the data for which participants had responded DKN, DKO, or TOT. The name given by the highest percentage of participants was considered the modal name. The percentage of participants who agreed on the modal name is the name agreement. In the case where two names had the same percentage of responses, the most specific name for the object was used (e.g. plastic cup as opposed to cup). Composite names in which the order of the words was rearranged (e.g. ham slice or slice of ham) were considered to be the same name.

#### H value

The H value for each object was computed. The statistic H is a value sensitive to the number and weight of alternative names. It is computed with the following formula [28]:
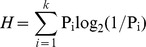
Where k refers to the number of different names given to each picture and excludes the DKN, DKO, and TOT responses, and 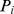 is the proportion of participants that gave a name for each object. This proportion varies across pictures because of the exclusion of the DKN, DKO, and TOT responses. The H value of a picture with a unique name and no alternative is 0. The H value of a picture with two names provided with an equivalent frequency is 1.00. This value is smaller for an alternative that is provided to a lower frequency rate. On the other hand, the H value increases as a function of the number of alternatives. For instance, one picture with its modal name provided by 50% of participants and two alternative names each with a frequency of 25% would have an H value of 1.50.

#### Modal category and category agreement

The modal category and category agreement were computed following the same procedure used for the names. These statistics were computed on the first category selected by the participants. A second category was rarely selected by participants and was considered only when two or more categories were selected at the same frequency for a stimulus. However, the second response was not added to the percentage of agreement.

#### H_cat_ value

An H value for the category, referred to as a 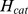 value, was measured following the same procedure used for the names.

#### Variables rated on a 5-point scale

Familiarity, visual complexity, object agreement, viewpoint agreement, and manipulability were computed by averaging the scores on the 5-point rating scale and by calculating the standard deviations.

#### Statistical analyses

Means and standard deviations were analyzed using independent sample t-tests, with the stimuli as for the participants and the categories as for the between-“stimulus” variables. Comparisons of categories were limited to the most commonly used and studied categories in cognitive science. The categories included animal, food, tool, musical instrument, weapon and vehicle. Tool, musical instrument, weapon, and vehicle are typically used as non-living or man-made concepts and are generally opposed to food and animal that are used as living or natural concepts. Because many food items of the BOSS are non-living (e.g. bottle of wine), a second category of food was created for the analyses which included only fruits, vegetables, and nuts. Categories analyzed thus consisted of animal (i.e. all animals collapsed together, except for the mussel, the seashell, and the fish skeleton), food, hand labour tool and accessory, musical instrument, vehicle, and weapon and war related item, as well as a seventh category including fruit/vegetable/nuts. Category comparisons were done for all norms except H value, H_cat_ value, and TOT, in order to reduce the number of comparisons. Alpha threshold was Bonferroni corrected to .00003 for multiple (189) comparisons.

Comparisons between genders were also performed with independent sample t-tests. Samples opposed stimuli responded by males and by females. Gender differences were examined for the mean norms and within each of the seven categories retained for the analyses. Alpha threshold was Bonferroni corrected to .0042 for multiple (12) comparisons.

## Results

### Norms

Table 1 summarizes the agreement and ratings obtained for each normative dimension. The stimulus-specific norms are presented in supporting Tables S1 and S2. In these tables, photo stimuli are sorted as a function of their filename, which at times, differs from the modal name and is more precise. All norms except those related to category are listed in Table S1. Categories, category agreements and H_cat_ for all stimuli, including the 538 photos of phase I, are presented in Table S2.

**Table 1:** Norms.

	Male (n = 20)			Female (n = 22)		Gender
Normative dimension	Mean	SD	SD	Mean	SD	comparison
Modal Name Agreement*	58%	25%	25%	61%	26%	t = 2.240***
*H* value*	1.89	1.06	0.97	1.53	0.09	t = 4.255***
DKO	3%	7%	6%	4%	10%	t = 4.903***
DKN	5%	8%	8%	6%	9%	t = 4.115***
TOT	3%	4%	3%	4%	7%	t = 12.458***
Category Agreement*	76%	21%	21%	77%	22%	t = 0.450
*H*_cat_ value*	0.97	0.75	0.72	0.87	0.75	t = 0.590
Familiarity	4.16	0.50	0.51	4.16	0.55	t = 0.147
Visual Complexity	2.43	0.54	0.52	2.49	0.59	t = 4.905***
Object Agreement**	3.69	0.52	0.54	3.57	0.57	t = 4.799***
Viewpoint Agreement**	3.60	0.44	0.45	3.45	0.51	t = 8.240***
Manipulability	2.57	0.78	0.84	2.67	0.76	t = 4.527***

*The modal name and category of males and females were not systematically the same, thus explaining why the norms of all subjects do not correspond to the averages of the two subgroups.

**Statistics for 464 stimuli.

***p<.0001.

### Norms per categories

The norms for each category, computed for all 1468 photos of the BOSS, are presented in Table 2. The first comparisons of categories, carried out on the categories in the upper part of Table 2, were conducted to determine whether some types of concepts were more difficult to recognize or name than others. DKO was significantly higher for tools (t(201) = 5.150, p<.00001) and weapons (t(201) = 5.150, p = .00003) than for animals. Tools were more difficult to name than all categories (all p<.00003) except musical instruments.

**Table 2:** Norms as a function of categories.

Category	Nb BOSS 2010	Nb BOSS 2014	Total Nb	DKO	DKN	TOT	NA	*H*	CA	Hcat	Fam	VC	OA	VA	Manip
Food	93	75	168	4%	4%	1%	63%	1.68	92%	0.38	4.24	2.25	4.05	3.89	1.96
- Fruit, vegetable & nut*	59	15	74	3%	5%	2%	74%	1.19	97%	0.20	4.28	2.25	4.27	3.99	1.66
Hand labour tool & accessory	49	46	95	7%	14%	4%	53%	2.15	70%	1.20	4.03	2.37	3.85	3.67	2.85
Musical instrument	5	40	45	4%	9%	4%	62%	1.65	89%	0.52	4.07	2.60	3.97	3.74	3.58
Vehicle	3	61	64	1%	5%	3%	56%	2.02	76%	0.97	4.24	2.74	3.60	3.40	2.59
Weapon & war related item	1	35	36	6%	6%	2%	59%	1.90	76%	1.04	3.73	2.24	3.65	3.56	2.90
Animal**	1	141	142	1%	5%	2%	71%	1.23	89%	0.49	4.02	3.09	3.77	3.50	1.89
- Bird	0	32	32	1%	5%	1%	72%	1.22	97%	0.18	4.01	3.16	3.74	3.46	1.85
- Canine	0	8	8	1%	6%	1%	63%	1.65	82%	0.70	4.20	3.00	3.46	3.45	2.38
- Crustacean	2	6	8	5%	7%	4%	54%	1.81	68%	1.32	3.88	3.13	3.57	3.69	1.88
- Feline	0	10	10	0%	2%	2%	62%	1.64	78%	0.93	4.27	3.10	3.83	3.58	2.06
- Fish	0	16	16	2%	7%	1%	75%	1.14	78%	0.94	3.71	3.02	3.44	3.49	1.74
- Insect	0	13	13	1%	1%	1%	73%	1.12	92%	0.40	4.18	3.06	3.86	3.38	1.82
- Mammal	0	42	42	1%	4%	2%	76%	1.00	93%	0.31	4.15	3.07	3.98	3.64	1.94
- Reptile	0	9	9	0%	3%	1%	60%	1.34	95%	0.29	3.98	3.21	3.31	3.34	1.98
- Sea mammal	0	7	7	2%	5%	2%	67%	1.49	76%	0.96	4.03	2.86	4.10	3.36	1.67
Body part	0	18	18	0%	0%	0%	69%	1.29	82%	0.78	4.67	2.57	4.01	3.64	3.14
Building infrastructure	0	96	96	3%	7%	4%	54%	2.17	67%	1.36	4.23	2.26	3.48	3.65	2.21
Building material	15	12	27	6%	11%	4%	49%	2.13	57%	1.64	4.35	2.18	3.81	3.60	2.19
Clothing	33	31	64	2%	3%	1%	63%	1.70	83%	0.70	4.13	2.18	3.65	3.56	2.93
Decoration & gift accessory	31	49	80	3%	6%	2%	56%	1.99	68%	1.24	4.05	2.52	3.45	3.61	2.21
Electronic device & accessory	44	47	91	4%	5%	2%	50%	2.30	68%	1.28	4.13	2.64	3.77	3.62	2.64
Furniture	3	32	35	1%	2%	2%	58%	1.87	78%	0.91	4.49	2.06	3.39	3.66	2.95
Game, toy & entertainment	27	40	67	3%	7%	2%	52%	2.15	76%	0.94	4.24	2.32	3.78	3.66	2.72
Household article & cleaner	38	21	59	2%	6%	2%	57%	2.02	55%	1.77	4.26	2.21	3.81	3.62	2.70
Jewel & money	7	1	8	2%	1%	1%	77%	1.07	60%	1.34	4.37	2.57	3.75	3.75	3.09
Kitchen & utensil	71	46	117	3%	8%	2%	53%	2.19	80%	0.88	4.23	2.23	3.86	3.78	2.62
Medical instrument & accessory	12	2	14	5%	9%	3%	57%	1.99	67%	1.38	4.24	2.41	3.94	3.73	3.11
Natural element	9	31	40	2%	3%	2%	65%	1.52	82%	0.74	4.14	2.61	3.56	3.66	2.02
Outdoor activity & sport item	19	75	94	4%	5%	3%	61%	1.73	72%	1.14	4.19	2.29	3.86	3.74	3.26
Skincare & bathroom item	34	11	45	2%	4%	2%	65%	1.60	74%	1.11	4.16	2.25	3.90	3.57	3.38
Stationary & school supply	42	18	60	1%	5%	3%	59%	1.87	75%	1.05	4.29	2.12	3.98	3.62	2.63

Nb = Number of stimuli, DKO = Don’t know object, DKN = Don’t know name, TOT = Tip-of-the-tongue, NA = Modal name agreement, H = H value, Fam = Familiarity, VC = Visual Complexity, CA = Category Agreement, Hcat = H value for category, OA = Object Agreement, VA = Viewpoint Agreement, Manip = Manipulability.

*Fruit, vegetable, and nut category was statistically compared with the other categories.

**All animals were collapsed together and compared statistically with the other categories. Subgroups of animals were not statistically compared.

The next comparisons looked at differences of modal name and category agreement. Animals and fruits/vegetables/nuts were named with a relatively similar consensus and their modal name agreement was significantly higher (all p<.00003) than that for tools and vehicles, which yielded more inconsistent names. The modal name agreement for fruits/vegetables/nuts was also significantly higher than for foods (t(166) = 5.326, p<.00001). The lower name agreement for tools was contingent to the lowest category agreement. Tools were classified more inconsistently than animals, foods, fruits/vegetables/nuts, and musical instruments (all p<.00001). In contrast, fruits/vegetables/nuts were classified more consistently than all other categories (all p<.00001), except musical instruments.

The least familiar category was that of weapons. They were significantly different from vehicles, foods, and fruits/vegetables/nuts (all p<.00001). The most complex stimuli were animals which were rated significantly higher than all other categories (all p<.00001). Vehicles were also more visually complex than foods, fruits/vegetables/nuts, and weapons (all p<.00001). Finally, musical instruments were more complex than foods (t(211) = 5.907, p<.00001) and fruits/vegetables/nuts (t(117) = 5.127, p<.00001).

Object agreement was the highest for fruits/vegetables/nuts, meaning that the photos in this category matched the mental image evoked by the concepts to a larger extent than the other stimuli. Object agreement for foods was significantly higher than all other categories except for musical instruments (all p<.00001). Viewpoint agreement was also the greatest for fruits/vegetables/nuts and for foods in general. These categories had a viewpoint agreement significantly higher than animals and vehicles (all p<.00001).

Finally, large differences were found with respect to manipulability. Foods, fruits/vegetables/nuts, and animals had manipulability ratings that were significantly smaller than all other categories (all p<.00001). In addition, musical instruments, which had the highest rating, were significantly more manipulable than vehicles (t(107) = 7.789, p<.00001).

### Norms per sex

Norms of males and females and the statistics resulting from their comparisons are presented in Table 1. Modal name agreement, DKO, DKN, and TOT were all significantly higher in females than in males. Females also rated visual complexity and manipulability with higher scores than males. In contrast, males provided significantly higher scores for object and viewpoint agreement than females. No differences were denoted for category agreement and familiarity.

Table 3 presents the norms of males and females within seven categories. Although they were not systematically significant, differences between genders were consistent with those described in Table 1, except for the tool category. Tools were more familiar to males and named with a higher agreement. Tools were the items that females recognized and named with the greatest difficulty, compared to males. Those difficulties also occurred for weapons, despite a greater modal name agreement for females.

**Table 3:** Norms as a function of gender.

Category	Gender	DKO	DKN	TOT	NA	*H*	CA	Hcat	Fam	VC	OA	VA	Manip
Food	Male	5%	3%	1%	54%	1.82	89%	0.46	4.29	2.23	3.81	3.86	2.08
(n = 75)	Female	4%	4%	2%	57%	1.63	92%	0.32	4.28	2.23	3.68	3.61	2.30
Fruit/vegetable/nut	Male	5%	5%	1%	52%	1.85	94%	0.28	4.27	2.21	3.91	4.18	1.86
(n = 15)	Female	4%	5%	4%	63%	1.43	98%	0.12	4.30	2.28	3.66	3.58	2.06
Tool	Male	3%**	8%**	1%**	62%*	1.67	74%	1.01	4.11*	2.24	3.84	3.84*	3.00
(n = 46)	Female	13%**	14%**	8%**	52%*	1.86	72%	1.07	3.72*	2.42	3.69	3.53*	2.83
Musical instrument	Male	4%	7%	2%**	64%	1.46	90%	0.46	4.01	2.47	4.09	3.89**	3.63
(n = 40)	Female	4%	10%	6%**	62%	1.41	91%	0.38	4.03	2.68	3.81	3.41**	3.62
Vehicle	Male	0%	5%	1%**	57%	1.82	75%	0.88	4.24	2.69	3.73*	3.59**	2.46
(n = 61)	Female	2%	7%	5%**	63%	1.52	79%	0.83	4.21	2.80	3.42*	3.21**	2.69
Weapon	Male	2%*	3%*	1%**	55%	1.87*	77%	0.98	3.86	2.18	3.88*	3.76*	3.05
(n = 35)	Female	10%*	9%*	4%**	65%	1.37*	77%	0.88	3.60	2.22	3.47*	3.34*	2.77
Animal	Male	1%	5%	1%*	70%	1.22	89%	0.45	3.94**	2.90**	3.88**	3.64**	1.70**
(n = 141)	Female	2%	4%	2%*	72%	1.06	89%	0.44	4.11**	3.24**	3.66**	3.37**	2.13**

DKO = Don’t know object, DKN = Don’t know name, TOT = Tip-of-the-tongue, NA = Modal name agreement, H = H value, Fam = Familiarity, VC = Visual Complexity, CA = Category Agreement, Hcat = H value for category, OA = Object Agreement, VA = Viewpoint Agreement, Manip = Manipulability.

*The agreement or rating is significantly different (.05< p <.0042) from the agreement or rating of the other gender.

**The agreement or rating is significantly different (p<.0042) from the agreement or rating of the other gender.

### Correlations

As is generally done in normative studies, the relation between the different normative dimensions was examined using correlational analyses. The alpha threshold was Bonferroni corrected and lowered to .0014. Results, which are presented in Table 4, show that the strongest correlations were between the agreement (name and category) and their respective H value. Name agreement correlated with all other norms except for visual complexity. In Brodeur and colleagues [15], modal name agreement did not correlate with category agreement however, in the present study there was a weak but significant correlation.

**Table 4:** Matrix of correlations.

	NA	H	Fam	VC	CA	Hcat	OA	VA
H	−.952*							
Fam	.340*	−.384*						
VC	.046	−.056	−.338*					
CA	.166*	−.194*	.081	.247*				
Hcat	−.172*	.203*	−.113	−.270*	−.960*			
OA	.288*	−.352*	.354*	−.034	.095	−.096		
VA	.176*	−.168*	.168*	−.097	.006	−.006	.323*	
Manip	.198*	−.223*	.445*	−.349*	.019	−.033	.183*	0.124

NA = Modal Name Agreement, Fam = Familiarity, VC = Visual Complexity, CA = Category Agreement, OA = Object Agreement, VA = Viewpoint Agreement, Manip = Manipulability.

*Significant correlation.

Object agreement and viewpoint agreement also exhibited a pattern of results very similar to that found in Brodeur and colleagues [15]. These ratings correlated with name dimensions and familiarity but not with category dimensions and visual complexity. The normative dimensions that differ the most between the present study and Brodeur and colleagues [15] are familiarity and visual complexity, which negatively correlated with each other in the present study. Moreover, familiarity no longer correlated with category agreement whereas visual complexity did. Finally, these two normative dimensions strongly correlated with manipulability.

## Discussion

This project proposes 930 new normative photos of concepts from different categories to be added to the 538 photos that already compose the BOSS [15]. The norms for the new set are very similar to those collected for the initial set, except for name agreement, which is slightly lower than in the initial set. This difference is essentially due to the use of more stringent criteria for keeping stimuli in Brodeur and colleagues [15], where only photos with a DKO below 20% and a name agreement above 20% were included in the analyses.

Some norms also differ from those of other normative sets of photos. For instance, Moreno-Martinez and Montoro [20] and Adlington and colleagues [4] had name agreement of 72% and 67%, respectively. The lower name agreement of the BOSS mostly pertains to its high number of stimuli and the inclusion of concepts that are necessarily more difficult to name. As argued in Brodeur and colleagues [15], adding new stimuli is generally associated with a reduction of name agreement. Rating for familiarity was higher than in Moreno-Martinez and Montoro [20] and Adlington and colleagues [4] as well as in most normative sets using line drawings. The BOSS includes a higher proportion of familiar everyday life objects (e.g. binder, pencil, toaster, etc.) than in these two other studies which, in contrast, offer a greater proportion of categories such as animals. Moreover, Adlington and colleagues [4] included concepts in their set that were intended to cover high, medium, and low familiarity ranges. Object and viewpoint agreements were very similar to the rating of typicality reported in Moreno-Martinez and Montoro [20] and visual complexity was only slightly smaller in the present study. Moreno-Martinez and Montero [20] also reported a higher rating for manipulability but the instructions were significantly different from those used in the present study.

The addition of animals, furniture, vehicles, weapons, musical instruments, and of many other types of concepts has not affected the mean ratings relative to Brodeur and colleagues [15] but it has slightly affected the pattern of correlations between norms. For instance, in contrast to Brodeur and colleagues [15], familiarity was negatively correlated with visual complexity. This negative correlation is consistent with most of the existing sets of images including a wide range of categories [28]. Moreover, manipulability was negatively correlated with visual complexity in the present study whereas this correlation was not significant in Brodeur and colleagues [15]. This new pattern of relationships is likely due to the addition of new categories in the present set. For instance, animals and vehicles, which were not in the original set, are amongst the most complex and the least manipulable concepts of the set. Moreover, the category of furniture was highly familiar but rated as visually simple, a pattern of correlation that contributes to the negative correlation found between familiarity and visual complexity. Overall, correlations found in this study are very similar to those reported in most previous studies using line drawings, likely because the present set includes animals, vehicles, furniture, and additional concepts also used in these other studies. For instance, like in other studies, name agreement correlated with familiarity [2], [8], [31]–[32] and norms of image agreement [2], [31], [33] but not with visual complexity [30]–[31], [33]–[37]. Accordingly, correlations between norms must thus be examined cautiously as they highly depend on the categories included in the set of stimuli and they may be relatively independent from the stimulus format.

By adding new categories of concepts and by increasing the number of stimuli per category, the present study demonstrated how the norms vary across different categories of concepts. Overall, it was found that animals are easily recognized and named and that they are consistently categorized within their specific sub-categories (i.e. bird, reptile, mammal, etc). Animals are also the most visually complex, most likely due to furs and feathers that represent a rich texture. Most animals in the present set are common but there are also unfamiliar animals such as a fennec, a cuttlefish, and a horseshoe crab. This contributed to increase DKO responses and decrease the familiarity rating. Moreover, some animals were confounded with similar animals, such as the alligator which was recognized as a crocodile, the caribou as a moose, and the falcon as an eagle.

The food category also has distinctive features. Food, and more particularly fruits, vegetables, and nuts are among the concepts that are the easiest to recognize and name, along with the fact that they are also among the most familiar concepts. Fruits, vegetables, and nuts are also the least manipulable in the sense that they are not associated with specific manipulations that allow distinguishing among them. Finally, foods obtained the highest object agreement, which suggests that the BOSS pictures were very consistent with the way people imagined these concepts. This is probably due to the fact that most food items, including fruits, vegetables, and nuts are not man-made, and therefore, are less subject to various designs.

The tool category includes concepts with heterogeneous features which led to a large variability along the different dimensions. There are familiar tools (e.g. leaf rake) which are easily named, categorized, and associated to specific uses and there are unfamiliar tools, such as professional tools (e.g. flooring stapler) which are difficult to name and use. Moreover, category agreement was lowered because some tools can be used for multiple purposes that can be related to another category (e.g. ice scrapper, metal brush, etc.). This heterogeneity across tools calls for caution when interpreting norms and reminds that a mean is not warrant of the individual components of some categories.

For some categories, differences between genders were to be expected. For instance, previous studies showed a naming advantage for females with living things and a naming advantage for males with non-living things [38]–[39]. Comparisons of genders indicate that females had more difficulty recognizing and naming tools and weapons than males. Tools and weapons were also less familiar to females, although this difference was significant only for tools. This can be explained by a lower interest or use of these types of objects by females in general. Tools and weapons were the only categories with an atypical pattern of gender differences. The typical pattern, found in most categories, consisted in a greater use of the DKO, DKN, and TOT by females, in addition to a higher modal name agreement and a lower H value. Instead of reflecting a naming difficulty, the higher rate of DKO, DKN, and TOT in females could indicate that they tend to avoid giving a name when they think this name is incorrect. This tendency necessarily reduces the variability of names and increases the modal name agreement. Females also rated visual complexity and manipulability with higher scores. The two genders reach comparable agreement when categorizing concepts and rate familiarity similarly. On the other hand, object and viewpoint agreements were higher in males. This could simply be explained by the fact that most photos were selected and taken by a male (i.e. first author).

Norms are fundamental not only to characterize stimuli but also to measure variables that could introduce confounding effects. Confounding effects were demonstrated several times. For instance, Laws and Neve [40] compared living and non-living stimuli and showed that the disadvantage in naming living stimuli was reversed after controlling for familiarity, visual complexity, and name frequency. Similar findings were replicated with other categories and stimulus dimensions [12], [41], which led Laws [42] to conclude that: “it is necessary to examine the performance of controls on sets of living and nonliving stimuli that are not confounded by these and other potential artefactual variables” (p. 842). In another study, Fillitier and colleagues [43] reported shorter response times for non-manipulable items compared to manipulable items. When they controlled for familiarity by including only familiar items in their analyses, they obtained the opposite effect. These confounding effects do not discard the existence of an effect inherent to the categories but they underline the importance of fully characterizing the stimuli before drawing conclusions on an effect.

## Conclusion

Norms are a precious asset that should be considered when creating experimental conditions in order to control for potential confounding effects. The BOSS now includes 1,468 normative colored photos of various concepts from multiple categories. The BOSS also offers 1,179 non-normative photos depicting other exemplars of the normative concepts, and the normative concepts photographed from different viewpoints. In addition, 275 photos are also available in a black and white line drawing version. Norms collected thus far for the BOSS include those described in the present study as well as norms related to symmetry [44]), color diagnosticity (unpublished), and different actions afforded by the objects including those for grasping, using, and moving the object [45]–[47]. There are yet no norms on the names of the concepts, such as frequency and age-of-acquisition, but they may be collected in the future. Finally, norms were collected from English native speakers [15] and French native speakers [48]. More information about the BOSS can be found at http://sites.google.com/site/bosstimuli/.

## Supporting Information

Table S1 **List of all stimulus-specific norms, except category agreement and Hcat.**
(PDF)

Table S2 **List of all stimulus-specific norms of category agreement and Hcat.**
(PDF)
