# Supplementary material for: Bank of Standardized Stimuli (BOSS) Phase II: 930 New Normative Photos
Source: PLoS One. 2014 Sep 11;9(9):e106953. doi: 10.1371/journal.pone.0106953 (PMC4161371; doi:10.1371/journal.pone.0106953)
Supplement: Table S1 — List of all stimulus-specific norms, except category agreement and Hcat. (PDF) [file pone.0106953.s001.pdf]

Filename	DKO	DKN	TOT	Modal name	NA	H	Fam	VC	OA	VA	Manip
**8ball**	0%	0%	2%	8 ball	49%	2.19	4.50	1.60			3.06
**accordion01**	0%	5%	17%	Accordion	97%	0.20	4.26	3.33	4.03	3.84	4.12
**aceofdiamond**	0%	0%	0%	Ace of Diamond	26%	2.65	4.76	1.33			3.03
**acorn**	0%	5%	2%	Acorn	79%	1.07	4.29	2.29	3.94	3.78	1.38
**acousticguitar02**	0%	0%	0%	guitar	71%	1.10	4.52	2.45	3.94	3.69	4.71
**addressplate**	0%	5%	5%	Address	18%	3.52	4.40	1.67			1.82
**africanelephant**	0%	0%	0%	elephant	95%	0.28	4.48	2.95	4.34	3.69	2.68
**aircompressor**	29%	26%	5%	Air compressor	35%	2.82	2.50	3.41			1.59
**airconditioner**	2%	0%	5%	air conditioner	53%	2.45	4.50	2.85			2.39
**airhockeytable**	0%	2%	5%	air hockey table	79%	1.24	4.40	2.48	4.19	3.34	3.38
**airvent**	0%	2%	7%	Vent	50%	2.44	4.29	1.93			1.68
**alarmsystem**	7%	7%	5%	alarm system	21%	3.58	4.14	2.79			2.68
**alligator**	0%	0%	0%	Crocodile	55%	0.99	4.02	3.10	4.31	3.50	1.94
**aloe01**	0%	5%	0%	Plant	50%	2.45	4.10	2.38			1.79
**aluminiumfoil**	0%	2%	0%	Aluminum foil	46%	2.22	4.79	1.88			2.65
**americangoldfinch**	2%	7%	0%	bird	79%	1.16	3.76	2.95	3.31	3.69	2.09
**americanwhitepelican**	0%	5%	2%	pelican	59%	1.59	3.81	3.31	3.61	3.89	1.56
**amphora**	7%	17%	0%	Vase	29%	3.60	3.37	2.50			1.50
**anchor**	2%	2%	0%	Anchor	93%	0.45	4.20	1.85	3.81	2.72	2.32
**angelstatue**	0%	2%	0%	Angel statue	41%	2.16	3.57	2.98			1.62
**ant**	0%	0%	0%	Ant	86%	0.82	4.62	2.67	3.91	3.47	1.65
**antelope**	0%	12%	0%	antelope	32%	2.30	3.67	2.95			1.47
**antlers**	0%	2%	0%	Antlers	73%	1.49	4.02	2.45	4.34	4.16	1.97
**apron**	0%	0%	0%	apron	95%	0.32	4.57	1.93	3.25	4.00	3.24
**aquarium**	0%	0%	0%	aquarium	57%	1.46	4.29	2.86	2.81	4.03	1.97
**arm**	0%	0%	0%	arm	71%	1.47	4.93	1.98	4.22	3.91	3.47
**armadillo**	5%	7%	10%	Armadillo	88%	0.71	3.95	3.41	3.84	3.69	1.33
**armchair02**	0%	2%	0%	chair	56%	2.15	4.62	2.31	2.06	3.72	3.26
**arrow02**	0%	2%	0%	arrow	93%	0.38	4.24	1.71	4.06	3.94	3.53
**artichoke01b**	0%	24%	17%	Artichoke	64%	1.60	3.98	2.76			1.67
**asianelephant**	0%	0%	0%	elephant	93%	0.44	4.62	3.31	4.22	4.00	2.53
**athleticsock**	0%	0%	0%	sock	81%	1.16	4.64	1.60	3.69	3.25	3.82
**atm**	0%	0%	0%	Atm	48%	1.58	4.71	2.74			3.74

**audiocablesplitter**	7%	12%	5%	Cable	9%	4.45	3.95	1.78			1.85
**awning**	0%	33%	14%	Awning	59%	1.91	4.15	1.90			1.85
**backfloat**	44%	10%	2%	Floatation device for child	11%	3.95	2.73	2.39			2.28
**bacon**	0%	5%	2%	Bacon	77%	1.01	4.40	2.05	3.78	4.03	2.00
**badger**	2%	24%	7%	Badger	54%	2.37	3.22	3.37			1.53
**badmintonracket**	0%	0%	0%	Badminton Racket	76%	1.12	4.33	2.05	4.31	4.00	3.94
**balalaika**	10%	33%	2%	Guitar	30%	2.87	2.93	2.54			2.97
**balcony02**	5%	2%	2%	Balcony	71%	1.74	4.20	2.39	3.16	3.58	1.79
**ballofstring**	5%	10%	10%	string	31%	3.20	3.95	2.02			2.18
**banjo**	0%	7%	5%	banjo	95%	0.36	4.07	2.79	4.09	3.03	3.62
**barbecue03**	0%	0%	0%	Barbecue	36%	2.34	4.81	3.02			3.38
**barbecuelighter**	2%	7%	7%	lighter	54%	2.40	4.21	2.24			2.97
**barbedwire01**	0%	7%	2%	barbed wire	68%	1.50	4.05	2.55	3.84	3.97	2.06
**barn**	0%	0%	2%	Barn	71%	1.68	4.00	2.55	3.53	3.75	1.47
**barnowl**	0%	0%	2%	owl	98%	0.17	4.43	3.33	4.06	3.94	1.91
**barrel01**	0%	0%	0%	barrel	86%	0.87	4.14	2.07	4.13	4.09	1.76
**baseballbat**	0%	0%	0%	baseball bat	81%	0.89	4.76	1.69	3.41	3.59	4.38
**baseballglove**	0%	0%	0%	baseball glove	62%	1.48	4.33	2.40	3.31	3.73	3.91
**baseboard**	12%	14%	14%	molding	20%	3.40	3.83	1.86			1.48
**baseboardheater01**	17%	0%	5%	heater	42%	3.19	3.70	2.39			1.72
**basketball01**	0%	0%	0%	Basketball	95%	0.32	4.79	1.83	4.45	4.32	4.26
**basketballhoop01**	0%	0%	0%	Basketball hoop	38%	2.23	4.60	2.33			3.94
**bassethound**	0%	0%	0%	dog	60%	2.15	4.26	2.95	3.03	3.50	2.56
**bassguitar**	0%	0%	0%	guitar	45%	1.90	4.21	2.86			4.29
**bat**	0%	0%	0%	bat	95%	0.28	4.17	3.21	3.63	2.94	1.85
**bathtub**	0%	0%	0%	bathtub	83%	0.79	4.79	2.26	3.53	3.63	3.32
**battleaxe**	2%	5%	2%	axe	63%	2.23	3.83	2.26	2.57	3.83	3.09
**battleship**	2%	0%	2%	Battleship	38%	3.07	3.62	3.83			1.59
**bazooka**	38%	5%	2%	Bazooka	35%	3.13	2.76	2.45			2.26
**beachpaddle01a**	0%	10%	5%	Ping pong paddle	31%	2.81	4.00	1.69			2.94
**beachumbrella01**	0%	0%	5%	umbrella	58%	1.99	4.40	1.71	2.33	2.83	3.41
**beaver**	0%	7%	0%	beaver	100%	0.00	4.17	3.21	3.94	3.06	1.73
**bed**	0%	0%	0%	bed	81%	1.00	4.80	1.98	3.63	3.63	3.74
**beehive**	7%	2%	0%	beehive	63%	1.55	3.98	3.56	2.88	3.22	1.78

**beerbottle**	0%	0%	0%	beer bottle	88%	0.61	4.64	1.55	4.28	4.16	3.41
**belltower01**	7%	12%	2%	Church tower	21%	3.35	3.71	3.12			1.88
**belugawhale**	0%	2%	5%	beluga whale	51%	2.22	4.02	2.67			1.41
**bench01**	0%	2%	0%	bench	76%	1.37	4.33	1.71	2.91	3.39	2.71
**beret01**	29%	0%	5%	Beret	54%	2.16	3.61	1.70			1.81
**bicycle**	0%	0%	0%	Bike	43%	1.74	4.83	2.88			4.18
**bikelock**	0%	5%	12%	bike lock	57%	1.84	4.52	2.07			2.85
**bikepump01**	0%	2%	0%	Air pump	37%	2.34	4.52	1.88			3.65
**bikerack**	12%	19%	7%	Bike rack	19%	4.02	3.86	2.10			2.25
**bikewheel**	0%	0%	0%	Bike wheel	26%	2.63	4.67	2.26			2.82
**birdfeeder**	5%	2%	0%	Bird feeder	82%	1.08	4.07	2.69	3.72	3.56	1.94
**birdhouse**	0%	0%	2%	Bird House	93%	0.44	4.29	2.43	3.41	3.28	1.82
**birdnest**	0%	0%	0%	Birds nest	71%	1.05	4.38	2.83	4.31	3.63	1.85
**birdseeds**	31%	5%	2%	Bird food	19%	3.42	2.83	3.10			1.50
**birthdaycandle**	0%	0%	0%	candle	52%	1.00	4.69	1.83	2.90	2.72	3.35
**bison**	0%	12%	2%	buffalo	64%	1.56	3.95	3.17	4.28	3.88	1.62
**blackbear**	0%	0%	0%	Bear	50%	1.36	4.33	3.07			2.09
**blackolive**	2%	0%	2%	Olives	60%	1.74	4.48	2.05	3.06	3.50	1.47
**bleachers**	0%	2%	10%	bleachers	70%	1.72	4.43	2.17	3.88	3.41	2.24
**blender**	0%	0%	0%	Blender	93%	0.48	4.52	2.29	3.53	4.13	2.79
**bluecheese**	0%	2%	0%	blue cheese	54%	1.40	4.02	2.40	3.91	3.31	1.91
**bluejay**	0%	2%	2%	blue jay	78%	0.99	4.17	3.38	4.50	4.31	1.50
**boat**	2%	2%	5%	boat	74%	1.35	3.95	2.57	2.88	3.10	2.21
**boatmotor**	33%	5%	2%	Boat motor	36%	2.91	3.00	2.93			2.42
**bolt01a**	0%	2%	10%	Bolt	57%	1.29	4.38	2.05			2.62
**boltcutter**	0%	12%	10%	Bolt cutter	33%	2.47	3.85	2.24			3.09
**boogieboard**	21%	17%	12%	Boogie board	43%	2.29	3.02	2.62			2.32
**bookshelf**	0%	0%	0%	Bookshelf	57%	1.63	4.74	1.60	3.88	4.06	2.65
**bottleofwhitewine**	0%	0%	0%	Wine bottle	36%	2.16	4.57	1.93			3.24
**bow**	2%	0%	0%	Bow	95%	0.33	4.20	1.76	3.59	3.09	4.29
**bowlingball**	2%	0%	0%	bowling ball	100%	0.00	4.43	1.95	3.48	3.81	4.06
**bowlingpin**	0%	2%	0%	bowling pin	100%	0.00	4.46	1.61	4.19	4.16	3.50
**bowlofcereal**	0%	7%	0%	Cereal	44%	1.89	4.48	2.40			2.09
**bowlofchips**	0%	0%	0%	bowl of chips	40%	2.26	4.81	2.21			2.41

**bowrake**	0%	5%	7%	rake	86%	0.77	4.48	1.75	3.47	3.75	3.79
**bowtie**	0%	0%	0%	Bowtie	88%	0.61	4.55	2.07	3.91	3.59	3.50
**boxingglove01**	0%	0%	0%	boxing glove	76%	1.11	4.24	2.21	3.53	3.69	3.74
**boxtrailer**	0%	21%	7%	Trailer	73%	1.57	4.07	2.29	2.67	3.67	1.82
**boxtruck**	0%	0%	0%	truck	76%	1.47	4.57	2.60	3.66	3.19	2.47
**brainmodel**	0%	0%	0%	brain	38%	2.49	4.19	2.74			2.35
**brassknuckle**	39%	10%	7%	Brass knuckles	78%	1.21	2.72	1.82			2.56
**brasslipstrike**	5%	40%	12%	Door bolt plate	11%	3.84	4.08	1.83			1.82
**bread**	0%	2%	0%	Bread	63%	1.86	4.34	2.39	3.22	3.75	2.03
**breadknife**	0%	0%	2%	knife	44%	1.92	4.62	1.76			3.24
**breadslice**	0%	0%	0%	Bread slice	36%	2.16	4.76	1.69			2.71
**bridge**	0%	0%	0%	bridge	90%	0.55	4.52	2.33	3.00	3.31	2.21
**bridgestick**	14%	33%	10%	Pool Rake	11%	4.06	3.35	1.64			2.91
**broadsword**	0%	2%	0%	sword	78%	1.46	3.86	1.93	3.56	4.19	3.21
**brownsugar**	26%	5%	2%	Brown sugar	86%	0.81	3.51	2.64	3.72	3.78	1.39
**bucket02**	2%	7%	0%	Bucket	50%	2.58	4.34	1.95			2.09
**buddhastatue**	0%	0%	0%	Buddha	45%	1.95	4.19	2.90			2.12
**buffaloskull**	7%	14%	5%	Bull skull	42%	2.95	3.31	2.98			1.35
**bull**	0%	0%	2%	Bull	88%	0.77	4.27	2.93	4.31	3.31	2.12
**bulldozer**	2%	7%	7%	Bulldozer	49%	2.39	4.12	3.37			2.29
**bullet**	0%	0%	0%	bullet	93%	0.48	4.07	1.71	4.06	3.03	2.88
**bulletbelt**	2%	2%	0%	Bullets	53%	2.53	3.90	2.36			2.38
**bumpercar**	0%	0%	7%	Bumper car	90%	0.68	4.33	2.90	4.00	3.22	2.53
**bun**	0%	0%	0%	Bread	29%	3.49	4.57	2.17			1.94
**buoy**	24%	21%	5%	buoy	62%	1.88	2.80	3.41			1.41
**bus**	0%	0%	0%	Bus	57%	1.85	4.69	2.45	3.13	3.25	2.29
**busshelter**	0%	0%	2%	Bus shelter	41%	2.54	4.69	2.05			2.15
**bust**	2%	2%	2%	Bust	33%	2.48	3.52	2.76			1.56
**butterfly**	2%	2%	0%	butterfly	85%	0.83	3.98	3.10	2.69	2.69	2.03
**butterknife**	19%	10%	7%	Butter knife	41%	2.52	3.73	1.86			2.14
**cabasa**	26%	38%	7%	shaker	33%	2.75	2.70	2.75			2.27
**cactus**	0%	0%	0%	cactus	93%	0.44	4.14	2.74	2.91	3.34	1.85
**cakeplatter**	0%	17%	2%	Cake platter	12%	4.10	3.71	2.29			2.03
**calendar**	0%	0%	0%	calendar	86%	0.85	4.74	1.98	3.59	4.13	2.44

**callbell**	0%	0%	2%	bell	80%	1.18	4.40	1.98	2.81	3.19	3.53
**campfire**	0%	0%	0%	fire	38%	2.47	4.33	2.93			3.09
**canadiangoose**	0%	0%	0%	canadian goose	38%	2.09	4.17	3.00			2.03
**candelabra**	0%	2%	0%	candelabra	37%	2.39	4.29	2.40			2.76
**candycane01a**	0%	0%	0%	candy cane	98%	0.16	4.90	1.69	4.28	3.19	2.44
**candydispenser**	0%	2%	5%	Candy dispenser	38%	2.33	4.62	2.95			3.03
**cannon**	0%	2%	2%	cannon	93%	0.50	4.26	2.52	3.88	3.66	2.65
**canoepaddle02**	2%	5%	0%	Paddle	59%	1.89	4.07	1.57	4.19	3.69	4.06
**cap03**	0%	2%	5%	hat	41%	3.04	4.21	2.21			2.79
**car**	0%	0%	0%	Car	81%	1.16	4.57	2.98	3.13	3.22	3.32
**carantenna**	43%	12%	2%	Antenna for a car	33%	3.04	2.64	2.00			1.68
**carbattery**	12%	17%	0%	Car battery	67%	1.76	3.59	2.51			2.16
**cardinal**	0%	10%	0%	bird	35%	2.59	3.90	3.27			1.53
**caribou02**	0%	5%	2%	Moose	64%	1.96	4.02	3.05	3.63	3.19	1.91
**carjack**	38%	21%	2%	Car jack	38%	1.94	2.40	2.63			2.71
**carlighter02**	40%	5%	2%	car lighter	45%	2.06	3.13	2.60			2.63
**carmat**	0%	5%	5%	car mat	39%	2.64	4.33	1.98			2.03
**carsidemirror01**	0%	0%	7%	car side mirror	23%	2.82	4.76	1.76			2.97
**cartonofeggs**	0%	0%	0%	Egg carton	45%	1.55	4.81	2.24			3.74
**casabamelon**	14%	10%	2%	squash	42%	2.26	3.95	1.79			1.66
**cashregister01**	0%	0%	2%	cash register	93%	0.49	4.48	2.67	3.75	3.13	3.29
**castle**	0%	0%	2%	castle	61%	2.38	3.79	3.55	2.88	3.53	1.55
**castletower**	0%	5%	2%	Castle tower	44%	2.17	3.76	2.74			1.55
**cat01**	0%	0%	0%	cat	88%	0.69	4.48	2.86	3.59	3.78	2.79
**catchermask**	5%	7%	0%	Catcher’s mask	16%	4.20	3.24	3.00			2.39
**cattail**	5%	24%	14%	Cat tail	21%	3.74	3.59	2.17			1.44
**cedarwaxwing**	2%	14%	0%	bird	83%	1.10	3.59	3.00	3.41	3.47	2.00
**ceilingfan01**	0%	0%	2%	Ceiling fan	71%	1.29	4.69	2.07	3.91	3.72	2.38
**ceilingspeaker**	14%	12%	0%	Speaker	48%	2.74	3.75	2.22			1.79
**cello**	0%	0%	10%	Cello	63%	1.66	4.26	2.67	4.38	3.84	4.06
**cementtruck**	0%	0%	5%	Cement truck	40%	1.87	4.45	3.26			2.00
**centurionhelmet**	0%	0%	2%	helmet	29%	3.38	4.05	2.86			2.15
**cessna**	0%	7%	0%	Airplane	38%	2.63	4.10	2.90			3.03
**chainlinkfence**	0%	0%	2%	fence	51%	2.08	4.52	1.95			2.53

**chainmail**	0%	10%	7%	Chain mail	37%	2.87	3.93	2.83			1.97
**chainsaw**	0%	5%	2%	Chainsaw	77%	1.25	4.38	2.71	4.22	3.53	3.88
**chair**	0%	0%	0%	chair	88%	0.76	4.83	1.64	3.00	3.81	3.71
**chameleon**	0%	19%	2%	Chameleon	42%	1.84	3.88	3.17			1.44
**chandelier**	0%	5%	2%	Chandelier	33%	2.44	4.38	2.48			2.09
**charcoalbarbecue**	12%	12%	2%	Barbecue	19%	3.26	3.75	2.28			2.38
**cheetah**	0%	0%	7%	cheetah	77%	1.25	4.31	3.31	4.09	3.84	1.79
**chefshat**	7%	2%	0%	Chefs hat	89%	0.70	3.81	2.05	3.34	3.91	2.24
**cherrypicker**	0%	40%	10%	Cherry picker	38%	2.55	3.71	2.98			2.15
**cherrytomato01**	0%	0%	0%	Tomatoes	40%	2.40	4.73	1.95			1.85
**chessknight03a**	0%	5%	2%	Chess piece	31%	2.82	4.52	2.14			2.65
**chest01**	2%	2%	2%	Chest	33%	3.03	3.90	2.15			2.00
**chickenshishkebab**	2%	5%	5%	ShishKabob	14%	3.68	4.10	2.36			2.21
**chiliflake**	17%	17%	0%	nuts	11%	4.28	3.30	2.90			1.91
**chime**	2%	31%	10%	chimes	67%	1.27	3.80	2.24			2.76
**chimney**	0%	0%	0%	chimney	86%	0.73	4.38	2.07	3.44	3.50	1.82
**chimpanzee**	0%	0%	0%	monkey	52%	1.75	4.31	3.26	3.32	3.53	2.32
**chipmunk**	2%	5%	7%	Chipmunk	67%	1.81	4.37	3.02	4.09	3.78	1.65
**chocolatechip**	10%	0%	0%	Hershey kisses	50%	2.07	4.45	1.76			1.53
**chocolatecroissant**	2%	10%	0%	Pastry	24%	3.57	3.98	2.29			1.82
**chocolatemilk**	0%	0%	0%	chocolate milk	52%	2.29	4.64	1.73	3.25	3.35	2.35
**christmaslights**	0%	0%	0%	Christmas lights	76%	1.03	4.64	2.07	4.25	3.53	2.47
**christmastree**	0%	0%	0%	Christmas tree	86%	0.77	4.71	3.14	3.84	4.16	2.85
**christmaswreath**	0%	0%	14%	wreath	53%	1.39	4.45	2.55			1.94
**church**	0%	0%	0%	church	81%	1.11	4.02	2.88	2.94	3.69	2.15
**cigar**	0%	5%	0%	Cigar	90%	0.68	4.12	1.70	4.34	3.75	3.91
**cinderblock**	0%	5%	0%	Cinder block	33%	2.65	4.57	1.83			1.88
**clarinet**	2%	17%	10%	Clarinet	47%	2.70	3.48	2.95			3.36
**cleaver02**	0%	0%	0%	Butcher knife	33%	2.10	4.45	1.74			3.44
**clothesdryingrack**	24%	12%	5%	Drying rack	24%	3.30	3.12	2.43			1.65
**cloud**	0%	0%	0%	cloud	95%	0.28	4.74	2.12	4.06	4.16	2.21
**clover**	0%	0%	0%	Clover	43%	2.25	4.36	2.12			1.71
**clownfish**	0%	2%	0%	Clownfish	46%	1.65	3.95	2.98			1.79
**coathook**	0%	2%	0%	Coat hook	37%	2.27	4.40	1.69			2.67

**coatrack**	0%	0%	2%	Coat rack	49%	2.22	4.55	1.69			3.35
**cobra**	0%	0%	0%	snake	71%	1.16	4.05	2.83	3.06	3.42	2.82
**cockroach02**	0%	0%	2%	Cockroach	46%	1.94	3.81	3.05			1.79
**cocktailshrimp02**	5%	2%	0%	shrimp	82%	0.89	3.95	2.64	3.47	3.66	1.88
**codedoorlock**	0%	10%	2%	Lock code	16%	4.11	4.29	2.48			3.29
**coffeemachine**	0%	0%	0%	coffee machine	71%	1.58	4.33	3.10	2.13	3.78	2.91
**coffeemaker**	0%	0%	0%	coffee maker	56%	1.34	4.63	2.61	3.90	4.16	3.03
**colonialhat**	14%	7%	0%	hat	58%	2.42	2.98	2.55			2.03
**coloringset**	0%	5%	0%	coloring set	15%	3.94	4.15	2.78			2.76
**column**	2%	14%	2%	columns	18%	3.98	3.62	2.93			1.24
**communitymailbox**	0%	5%	5%	mail box	74%	1.46	4.10	2.52	2.31	3.50	2.73
**compostbin**	26%	7%	5%	Composter	19%	3.69	3.18	2.30			1.78
**congadrum**	0%	12%	0%	drum	46%	2.53	3.98	2.38			4.09
**contactlens**	2%	2%	0%	bowl	58%	1.84	4.55	1.69	3.38	3.38	2.71
**convertible**	0%	0%	0%	Convertible	29%	2.69	4.33	2.69			3.59
**cookingpot**	0%	2%	5%	pot	64%	1.90	4.57	1.98	2.94	3.94	2.71
**cork02**	7%	2%	5%	cork	75%	1.06	4.33	1.57	4.22	3.28	3.00
**corkboard**	0%	7%	5%	Cork board	32%	2.81	4.52	1.69			2.65
**cormorant**	5%	10%	5%	bird	56%	2.08	3.21	3.19			1.56
**corn02**	0%	0%	0%	corn	64%	1.38	4.81	2.40	4.16	4.25	3.15
**cornet**	2%	10%	5%	trumpet	40%	3.01	3.86	2.95			3.64
**couch02**	0%	0%	0%	Couch	50%	1.75	4.81	2.12			3.21
**cougar**	0%	10%	10%	Cougar	35%	3.08	3.86	3.29			1.74
**cow**	0%	0%	0%	cow	93%	0.37	4.71	2.90	4.13	3.94	2.91
**cowbell**	12%	14%	0%	flask	61%	1.91	3.79	2.30			2.28
**crab01**	0%	2%	0%	crab	98%	0.17	4.12	3.10	3.78	3.28	1.79
**craneflower**	19%	5%	0%	flower	84%	0.93	3.24	2.71	2.68	3.58	1.55
**cremebrulee**	33%	5%	2%	Crème brulee	72%	1.48	3.21	2.64			1.54
**crocodile**	0%	0%	0%	Crocodile	48%	1.36	4.07	3.17			2.12
**cross01**	0%	0%	0%	cross	88%	0.76	4.55	1.38	4.16	3.94	2.91
**crosscountryboot**	5%	0%	2%	shoe	46%	2.62	3.26	2.40			2.61
**crosscountryski**	0%	7%	0%	Skis	79%	0.84	4.12	2.07	3.94	4.06	3.82
**crowdcontrolstanchion**	7%	29%	21%	Stanchion	11%	3.95	4.20	1.54			1.97
**crown**	0%	0%	2%	Crown	88%	0.72	4.43	2.74	4.06	4.25	3.44

**cruiseship**	0%	0%	0%	cruise ship	83%	0.96	4.38	3.74	4.22	3.69	1.76
**cupcake**	0%	0%	0%	cupcake	95%	0.32	4.81	2.26	4.00	4.28	2.41
**curlingiron01a**	2%	2%	0%	curling iron	48%	2.09	4.14	2.38			3.82
**curtain**	0%	0%	0%	curtain	86%	0.87	4.76	1.86	3.77	3.97	2.79
**cutlass**	0%	0%	0%	sword	74%	1.31	4.10	2.21	3.19	3.69	3.47
**cutleryset**	0%	2%	2%	cutlery set	30%	3.39	4.33	2.57			2.76
**cuttlefish**	7%	14%	0%	Squid	64%	1.96	3.24	3.36			1.29
**cymbal**	0%	17%	10%	Cymbals	81%	1.14	4.12	1.85	4.00	3.44	3.65
**daddylonglegs**	0%	2%	0%	Spider	88%	0.77	4.33	2.57	3.50	3.78	2.09
**daffodil**	0%	5%	5%	flower	76%	1.24	3.86	2.52	3.25	3.31	2.06
**daisy**	0%	2%	5%	flower	46%	1.53	4.02	2.57			2.50
**dalmatian**	0%	0%	0%	Dalmatian	71%	1.15	4.55	3.00	4.06	3.63	2.76
**dandelion**	2%	7%	0%	dandelion	61%	1.33	4.12	2.79	3.70	2.50	2.12
**dartboard**	0%	10%	0%	dart board	100%	0.00	4.46	2.76	4.41	4.28	3.74
**desk02**	0%	2%	2%	desk	50%	2.41	4.52	2.60			2.26
**desktopcomputer**	0%	0%	0%	computer	67%	1.86	4.88	2.86	3.69	3.81	4.24
**diaperbag**	0%	2%	0%	Bag	37%	3.11	4.19	2.17			2.62
**dinosaurskull**	0%	0%	0%	Dinosaur skull	31%	3.33	3.86	3.12			1.53
**diploma01**	0%	0%	0%	Diploma	33%	3.26	4.45	1.81			1.76
**diploma02**	2%	2%	0%	Diploma	55%	2.04	4.24	1.67	3.00	3.22	2.44
**discoball**	0%	0%	0%	disco ball	93%	0.48	4.55	2.60	4.38	3.88	2.44
**dishrack**	24%	21%	5%	Dish rack	38%	1.88	3.28	2.63			2.10
**djmixer01**	7%	7%	5%	Turntable	24%	4.17	3.73	3.32			3.67
**djmixer02**	7%	5%	2%	Dj turntable	8%	4.50	3.40	3.24			3.56
**doghouse**	0%	0%	2%	Dog House	98%	0.17	4.50	2.24	3.72	3.59	2.24
**dogsled**	0%	7%	0%	Dog sled	59%	2.15	3.76	3.29	3.39	3.39	2.59
**dolly01**	0%	31%	26%	Dolly	61%	2.06	4.24	2.05			3.24
**dolphin01**	0%	0%	0%	dolphin	98%	0.16	4.48	2.83	4.41	3.77	1.82
**donotentersign**	0%	2%	2%	Do not enter Sign	33%	3.05	4.50	1.43			1.94
**donotwalksign**	0%	0%	0%	Do not walk Sign	12%	4.60	4.81	2.36			2.79
**donut**	0%	0%	0%	Doughnut	95%	0.28	4.74	1.71	3.88	3.69	2.56
**doorbolt**	2%	14%	0%	lock	43%	2.54	4.02	2.50			2.44
**doorhandle**	0%	0%	0%	Door handle	50%	1.41	4.78	2.02			4.03
**doormat**	0%	0%	2%	Rug	29%	2.66	4.48	2.05			2.50

**dormer**	0%	7%	0%	window	82%	1.18	4.50	2.45	2.81	3.13	2.56
**doublebass**	0%	0%	14%	Cello	47%	2.35	4.00	2.79			4.09
**doubledoors**	0%	5%	0%	Doors	55%	1.99	4.45	2.21	2.89	3.78	3.09
**downhillski**	0%	0%	0%	skis	83%	1.07	4.17	2.05	3.94	3.66	3.97
**dragonfly01**	0%	5%	5%	Dragon fly	76%	1.38	4.12	3.26	4.03	3.28	1.74
**dreamcatcher**	2%	0%	0%	dream catcher	93%	0.49	4.05	2.64	4.22	4.44	1.88
**dresser02**	0%	0%	2%	Dresser	49%	2.34	4.55	2.19			2.82
**drink**	0%	2%	0%	drink	27%	3.42	4.10	1.90			3.06
**drinkshaker**	19%	21%	2%	Martini shaker	17%	3.24	3.46	2.00			2.68
**dromedary**	0%	0%	0%	camel	98%	0.16	4.21	2.71	4.00	3.75	2.18
**drumset**	0%	0%	0%	Drum set	64%	1.29	4.71	2.95	3.75	3.22	4.29
**drumstick**	0%	2%	0%	drum sticks	95%	0.33	4.55	1.57	3.91	3.88	3.97
**dryingmachine02**	0%	0%	0%	washing machine	50%	2.34	4.64	2.57			2.76
**duck01**	0%	0%	0%	duck	81%	1.00	4.31	3.00	3.84	3.47	1.85
**ducttape**	2%	0%	0%	Duct tape	73%	1.18	4.55	1.79	4.13	3.78	2.97
**dumpling**	43%	12%	0%	Dumpling	37%	3.11	2.39	2.50			1.52
**dumpster**	2%	0%	0%	Dumpster	37%	2.73	4.41	2.12			2.35
**dumptruck**	0%	0%	0%	truck	50%	1.49	4.50	3.21			2.38
**dvdcase**	2%	2%	2%	DVD case	36%	2.46	4.43	2.00			2.47
**eagle**	0%	0%	0%	eagle	67%	1.61	4.33	3.19	4.16	2.38	1.97
**ear**	0%	0%	0%	ear	83%	0.65	4.95	2.43	4.41	4.28	3.88
**eggnoodle**	0%	7%	0%	pasta	37%	2.85	4.24	1.93			2.21
**eggslicer**	19%	5%	2%	Egg slicer	26%	3.64	3.29	2.54			2.03
**eiffeltower**	0%	0%	0%	Eiffel tower	95%	0.32	4.62	3.29	4.56	4.38	1.82
**elbow**	0%	0%	0%	elbow	86%	0.77	4.90	1.88	4.09	3.75	3.44
**electricdrumset**	0%	12%	5%	Drum set	26%	3.09	3.79	3.14			3.26
**electricguitar01**	0%	0%	0%	Electric Guitar	62%	1.09	4.50	2.64	4.13	2.91	4.47
**electricitymeter**	12%	20%	10%	Hydro meter	29%	3.18	3.46	2.75			1.58
**electricmixer**	0%	2%	0%	mixer	54%	2.70	4.33	2.55	3.72	3.63	2.47
**electricoutlet**	0%	7%	0%	Electric outlet	28%	2.99	4.79	1.69			3.59
**electrictoothbrush01c**	0%	0%	0%	Electric toothbrush	69%	1.25	4.50	2.19	4.09	2.59	3.74
**electrictransformer**	33%	26%	7%	Transformer	21%	3.47	3.00	2.63			1.50
**ellipticalmachine**	0%	5%	7%	Elliptical machine	24%	3.23	4.33	2.74			3.53
**englishcucumber**	0%	0%	0%	cucumber	88%	0.69	4.74	1.86	4.41	4.19	2.06

**entrancegate**	0%	0%	2%	gate	71%	1.64	4.48	2.38	3.53	3.69	2.38
**escalator**	0%	0%	0%	Escalator	90%	0.60	4.81	2.64	4.28	4.22	3.00
**espressomachine**	10%	10%	5%	Espresso machine	53%	2.09	3.83	3.14			3.03
**exercisebike**	0%	12%	7%	Exercise bike	35%	3.19	4.10	2.98			3.56
**exercisemachine**	0%	5%	5%	exercise machine	42%	2.98	4.12	3.10			3.74
**exitsign**	0%	0%	0%	exit sign	95%	0.32	4.86	1.64	4.13	4.06	2.26
**eye**	0%	0%	0%	Eye	76%	1.07	4.90	2.98	4.26	4.23	3.74
**eyechart**	0%	10%	5%	eye chart	47%	2.92	4.50	2.31			2.94
**eyepatch**	2%	7%	0%	Eye patch	92%	0.52	3.86	1.50	4.47	3.97	3.15
**falcon**	2%	2%	0%	eagle	45%	2.38	4.00	3.39			1.94
**fanblades**	0%	2%	10%	fan	54%	1.53	4.17	2.02			2.21
**fanheater**	2%	5%	0%	fan	69%	1.64	4.26	2.33	2.50	3.41	2.00
**faucet**	0%	0%	12%	faucet	65%	1.91	4.71	2.02	3.28	3.66	3.82
**faxmachine**	0%	0%	0%	fax machine	62%	1.76	4.40	2.83	3.71	3.39	2.65
**fence02**	0%	2%	0%	fence	61%	1.67	4.38	2.24	3.13	3.48	1.94
**fennec**	0%	24%	0%	Fox	53%	2.09	3.31	3.10			1.56
**fern**	2%	0%	2%	Fern	53%	1.74	3.83	2.95			1.45
**ferry**	0%	0%	0%	Ferry	31%	2.85	4.10	3.55			1.85
**fighterjet**	0%	2%	0%	plane	24%	2.78	3.98	3.12			2.68
**file**	10%	21%	7%	File	58%	2.26	3.51	1.76			2.50
**filingcabinet**	0%	2%	5%	filing cabinet	64%	2.01	4.64	1.95	4.22	4.09	3.09
**fingernail02**	7%	2%	0%	Nail	50%	1.67	4.12	2.21			2.56
**fingerprint**	0%	0%	0%	finger print	93%	0.44	4.69	3.17	4.53	4.66	3.26
**firealarm**	0%	0%	0%	fire alarm	43%	2.28	4.50	1.79			2.21
**firehydrant**	0%	5%	2%	Fire hydrant	97%	0.17	4.60	2.21	3.97	2.94	2.50
**fireplace**	0%	0%	0%	Fireplace	86%	0.85	4.50	2.17	3.63	4.13	2.53
**firstaidkit**	0%	5%	0%	lunch box	33%	2.80	4.14	1.90			2.55
**firtree**	0%	0%	0%	Christmas tree	33%	2.63	4.64	2.95			2.56
**fishingrod**	0%	0%	7%	fishing rod	72%	0.98	4.24	2.38	3.81	3.84	3.97
**fishskeleton**	0%	0%	0%	Fish skeleton	67%	1.16	4.17	2.95	4.03	4.09	1.53
**flag**	0%	0%	0%	flag	71%	1.38	4.21	1.83	2.88	3.44	2.18
**flail**	10%	29%	5%	Mace	25%	3.20	3.41	2.49			2.26
**flamingo**	0%	0%	10%	flamingo	87%	0.81	4.43	3.17	4.34	4.06	1.97
**flintlockpistol**	2%	5%	0%	gun	44%	2.98	3.64	2.83			3.59

**flipflop01b**	0%	0%	0%	flip flop	60%	1.79	4.79	1.83	3.28	3.25	2.79
**floodlight**	14%	17%	2%	light	29%	3.23	3.45	2.76			1.82
**flooringstapler**	57%	14%	5%	Nail gun	20%	3.12	2.14	2.93			1.70
**floorlamp**	0%	0%	0%	lamp	86%	0.72	4.57	2.07	3.78	4.22	3.00
**floortile02**	0%	2%	0%	tile	61%	1.43	4.60	1.69	3.56	3.88	1.82
**flower01**	0%	10%	2%	flower	86%	0.83	3.71	2.83	2.91	3.06	1.88
**flowerwreath**	0%	14%	5%	wreath	32%	3.09	3.86	2.93			1.71
**flugglehorn**	0%	2%	5%	trumpet	64%	1.72	4.29	2.86	4.03	3.81	4.24
**fluorescentlightbulb**	0%	0%	0%	light bulb	67%	1.91	4.71	2.26	2.75	2.78	3.03
**foldingchair**	0%	0%	0%	folding chair	52%	1.13	4.76	1.88	3.06	3.39	3.44
**fonduefuel**	5%	21%	5%	Bottle	17%	4.32	3.55	2.19			1.77
**foosballtable**	0%	5%	12%	Foosball Table	68%	1.19	4.71	2.88	4.03	3.66	3.74
**foot**	0%	0%	0%	foot	69%	1.41	4.81	2.02	3.97	2.19	3.65
**football**	0%	0%	0%	football	98%	0.16	4.69	1.90	4.22	4.13	4.26
**footballhelmet**	0%	0%	0%	football helmet	79%	0.86	4.31	2.21	4.13	3.69	3.24
**footrest02**	2%	5%	17%	footrest	34%	2.39	4.44	2.05			2.62
**forklift**	0%	24%	10%	Forklift	68%	1.51	4.17	3.10			2.32
**fortunecookie**	0%	2%	0%	fortune cookie	98%	0.17	4.64	1.76	4.63	4.13	2.97
**freezer02**	2%	0%	0%	freezer	61%	2.15	4.38	1.79	3.97	4.00	2.42
**freighttruck**	0%	0%	0%	truck	50%	2.51	4.60	2.90			2.41
**frenchfries**	0%	0%	0%	fries	52%	1.27	4.93	1.95	4.03	3.97	2.71
**frenchhorn**	0%	12%	10%	French horn	45%	2.18	3.95	3.02			3.59
**fridge**	0%	0%	0%	Fridge	71%	1.16	4.88	1.76	4.22	4.16	3.32
**frisbee**	2%	5%	0%	Frisbee	95%	0.34	4.40	1.78	3.84	3.88	4.12
**funnel**	0%	5%	10%	funnel	97%	0.18	4.63	1.59	4.34	3.38	2.71
**fuse**	19%	10%	7%	Fuse	52%	2.53	3.41	2.65			2.14
**fusebox**	5%	19%	12%	Fuse box	22%	3.63	4.31	1.60			2.00
**gamecontroller01**	0%	0%	5%	Game controller	25%	3.37	4.55	2.00			3.56
**garbagebin**	0%	0%	0%	Recycling bin	43%	2.46	4.64	1.95			2.79
**garbagecan02**	0%	0%	0%	Garbage can	33%	2.90	4.57	1.93			3.12
**gardengnome02**	0%	7%	0%	Garden gnome	38%	2.19	4.00	2.48			1.47
**gardenswing**	0%	26%	14%	Garden swing	12%	4.10	4.12	2.45			2.56
**gardenutilityvehicle**	0%	26%	7%	tractor	18%	4.22	3.57	2.95			2.47
**garland**	10%	10%	12%	Tinsel	24%	3.09	4.05	2.24			1.94

**gasburner**	5%	10%	7%	burner	15%	4.09	4.17	2.54			2.29
**gastank**	0%	12%	7%	Gas jug	12%	3.84	4.21	1.86			2.44
**gavel**	0%	12%	10%	Gavel	58%	2.19	4.14	1.98			3.62
**gazebo**	2%	12%	10%	Gazebo	72%	1.69	3.76	2.79	2.94	3.59	1.50
**gearshift**	0%	5%	5%	Gear shift	32%	3.37	4.29	2.38			3.76
**gecko**	2%	7%	2%	Lizard	59%	1.86	3.52	3.31	3.00	3.75	1.38
**generator**	33%	14%	7%	Generator	42%	2.77	2.97	3.00			1.66
**gift01**	0%	0%	0%	Gift	48%	1.92	4.71	2.40			3.15
**giraffe**	0%	0%	0%	giraffe	100%	0.00	4.43	3.12	4.47	4.34	2.21
**giraffefigurine**	5%	0%	0%	Giraffe statue	23%	3.45	3.71	2.57			1.53
**goldnugget**	17%	2%	0%	rock	42%	1.95	3.22	2.78			1.68
**golfbag**	0%	2%	2%	Golf clubs	30%	2.83	4.14	2.93			3.97
**golfball**	0%	0%	0%	golf ball	98%	0.16	4.57	1.64	4.38	4.28	3.68
**gong**	2%	14%	7%	Gong	66%	1.91	3.73	2.40			3.26
**gorilla**	0%	0%	0%	gorilla	69%	1.44	4.14	2.98	3.78	3.97	2.32
**gps**	0%	7%	0%	GPS	62%	2.24	4.17	2.26	3.97	3.69	2.74
**graduationcap**	0%	5%	5%	Graduation cap	55%	1.71	4.36	1.93			3.00
**grandpiano**	0%	0%	0%	Piano	55%	0.99	4.45	3.57	4.06	3.97	4.65
**granolabar01**	0%	0%	0%	granola bar	88%	0.80	4.74	2.62	4.16	3.59	2.29
**grasshopper**	2%	2%	0%	Grasshopper	40%	2.08	3.62	3.40			1.68
**gravyboat**	0%	21%	2%	Gravy boat	47%	2.79	4.10	2.36			2.35
**greathornowl**	0%	0%	0%	owl	100%	0.00	4.31	3.36	3.66	3.78	2.00
**greatwhiteshark**	0%	0%	0%	shark	90%	0.53	4.33	2.86	4.19	3.56	2.12
**greyhound**	0%	2%	2%	dog	55%	1.62	4.21	2.98	2.84	3.19	2.26
**greywolf**	0%	0%	0%	Wolf	86%	0.85	4.24	3.07	4.34	3.50	2.03
**grill**	14%	5%	7%	grill	26%	3.26	3.60	1.59			1.82
**grizzly**	0%	0%	0%	Bear	64%	1.29	4.40	3.10	3.81	2.88	2.15
**guacamole**	10%	12%	5%	guacamole	39%	2.87	3.60	2.05			1.55
**guitarcase**	0%	0%	0%	Guitar case	98%	0.16	4.52	2.14	3.88	3.53	2.97
**gutteranddrainpipe**	0%	24%	17%	Gutter	32%	3.41	4.24	2.07			1.70
**gymnasticring**	14%	21%	7%	Gymnastic rings	38%	2.75	3.38	1.71			2.97
**halberd**	7%	19%	5%	axe	34%	2.97	3.20	2.33			2.28
**halogenlightbulb**	0%	7%	0%	light bulb	36%	2.96	4.02	2.33			2.15
**hammerheadshark**	0%	2%	7%	Hammerhead shark	68%	1.28	4.05	2.86	4.13	2.81	1.68

**hamslice**	0%	0%	0%	ham	33%	2.79	4.17	2.05			1.85
**hand01b**	0%	0%	0%	hand	81%	0.90	4.93	2.45	4.28	3.91	4.12
**handblender**	7%	7%	7%	Hand blender	24%	3.51	4.07	2.40			2.52
**handbrush02**	0%	5%	0%	brush	58%	2.35	4.10	2.07	2.78	2.94	2.91
**handcuffs**	0%	0%	0%	handcuffs	100%	0.00	4.48	2.24	4.25	3.88	3.91
**handgripper**	12%	45%	10%	Grip exerciser	7%	3.81	3.63	2.15			2.76
**handheldtelescope**	2%	10%	7%	telescope	79%	1.31	3.64	2.29	2.97	3.16	3.64
**handrail**	0%	7%	0%	Railing	51%	2.55	4.48	1.69			2.76
**handvacuum**	14%	7%	7%	Hand vacuum	27%	3.12	3.78	2.50			2.59
**hangar**	0%	21%	7%	Green House	23%	3.89	3.39	2.54			1.59
**hardhat02**	0%	0%	0%	Hard hat	36%	2.70	4.57	1.60			3.21
**harmonica**	0%	5%	0%	harmonica	100%	0.00	4.31	2.50	3.67	3.81	3.82
**harpoon**	5%	5%	0%	arrow	63%	1.55	3.78	1.80	3.47	3.94	2.82
**headlight**	0%	2%	0%	Headlight	39%	1.84	4.38	2.62			1.73
**heatpump**	14%	24%	7%	air conditioner	39%	3.20	3.29	2.74			1.64
**hedgeshears**	0%	17%	5%	Shears	30%	3.45	4.00	2.22			3.21
**hedgetrimmer**	12%	21%	2%	Hedge trimmer	26%	3.58	3.39	2.68			2.73
**helicopter**	0%	0%	0%	helicopter	90%	0.64	4.24	3.45	3.53	2.94	2.24
**helmet**	0%	2%	0%	helmet	39%	2.86	3.57	2.02			2.32
**hen**	0%	0%	0%	Chicken	45%	1.52	4.43	3.12			2.38
**heron01**	0%	7%	5%	bird	38%	2.84	3.88	3.05			1.53
**hibiscusflower02**	0%	10%	0%	flower	89%	0.70	3.81	2.55	3.25	3.34	1.91
**highstrikergame**	10%	55%	2%	Fair game	14%	3.66	3.37	2.59			3.32
**hihat**	7%	17%	5%	cymbals	23%	3.35	3.64	2.69			3.21
**hippopotamus**	2%	0%	0%	Hippopotamus	46%	1.46	4.24	2.93			1.62
**hockeygoalieglove**	21%	14%	5%	Goalie glove	24%	3.00	2.90	2.76			2.93
**hockeygoaliemask**	0%	0%	0%	hockey mask	19%	2.82	4.40	2.36			2.85
**hockeygoaliepad**	20%	2%	2%	Goalie pad	35%	3.23	3.57	2.34			2.97
**hockeypuck**	0%	0%	0%	hockey puck	83%	0.75	4.62	1.38	3.97	4.06	3.12
**hockeystick**	0%	0%	0%	hockey stick	93%	0.48	4.62	1.55	4.13	4.06	4.18
**hoe**	5%	24%	7%	hoe	41%	2.38	3.88	2.00			2.82
**holdfast**	0%	10%	0%	fish	89%	0.64	3.79	2.74	2.88	3.47	1.88
**honeybee**	0%	0%	0%	Bee	79%	1.04	4.67	3.31	4.03	3.31	2.29
**honeycomb**	2%	7%	2%	honey comb	65%	1.84	4.10	2.63	3.88	3.69	1.56

**horse**	0%	0%	0%	horse	100%	0.00	4.45	2.88	3.97	3.66	3.15
**horseshoe**	7%	5%	0%	Horseshoe	100%	0.00	3.98	1.88	3.25	3.09	2.68
**horseshoecrab**	29%	24%	10%	Stingray	25%	3.08	2.69	3.00			1.29
**hose**	0%	0%	0%	hose	50%	1.36	4.64	1.93			3.12
**hotdog**	0%	0%	0%	hot dog	57%	1.88	4.69	2.14	3.94	3.72	2.94
**hotdogweiner**	5%	2%	0%	hot dog	74%	1.38	4.24	1.79	2.94	3.75	2.28
**hubcap**	0%	7%	2%	hubcap	24%	3.56	4.14	2.38			2.00
**hulahoop**	0%	0%	0%	Hula hoop	93%	0.44	4.67	1.40	4.19	4.13	4.41
**humanskeleton**	0%	0%	0%	skeleton	52%	1.00	4.71	3.31	3.91	4.03	2.26
**humanskull**	0%	0%	0%	Human skull	48%	1.23	4.43	3.19			1.88
**huntingknife**	0%	0%	0%	knife	55%	2.45	4.19	2.12	3.28	3.81	3.53
**hyena**	10%	19%	5%	Hyena	68%	1.78	3.27	3.12			1.53
**icebucket**	17%	17%	0%	ice bucket	36%	3.34	3.54	1.93			1.84
**icecube02**	0%	0%	0%	Ice cube	74%	0.94	4.69	2.07	3.55	4.32	2.44
**icemaker**	12%	14%	2%	Ice box	27%	3.41	3.48	2.07			1.97
**icescraper**	5%	10%	2%	Ice scraper	46%	2.44	4.17	1.93			2.97
**inukshuk**	2%	14%	17%	Inukshuk	36%	2.53	3.76	2.80			1.56
**ipad02**	0%	0%	0%	Ipad	79%	1.10	4.60	1.76	4.25	3.44	3.41
**ipod**	0%	0%	0%	IPhone	40%	2.46	4.55	1.93			3.68
**ironingboard01**	0%	2%	5%	ironing board	79%	0.73	4.52	1.57	4.28	3.94	3.62
**jackhammer**	7%	14%	2%	Jackhammer	50%	1.81	3.59	2.98			3.29
**jackolantern**	0%	0%	0%	Jack o lantern	38%	2.12	4.71	2.43			2.65
**jackrabbit**	0%	0%	2%	Rabbit	88%	0.62	4.43	2.90	3.56	3.56	2.41
**jaguar**	0%	0%	0%	Leopard	57%	1.63	4.12	3.31	4.16	2.81	1.76
**jarofapplesauce**	0%	7%	0%	Apple sauce jar	26%	2.68	4.14	2.10			2.24
**jarofcapers**	17%	17%	0%	jar	29%	2.82	3.37	2.78			1.79
**jeans01**	0%	0%	0%	jeans	88%	0.76	4.67	1.86	4.06	4.00	3.56
**jeep**	0%	0%	0%	jeep	88%	0.64	4.64	2.95	3.88	3.16	2.85
**jellybean**	0%	0%	0%	jelly beans	98%	0.16	4.74	1.86	3.97	3.78	1.85
**jellyfish**	2%	7%	0%	Jellyfish	95%	0.35	3.71	2.66	2.97	3.50	1.66
**Jesusstatue**	0%	0%	0%	Jesus statue	43%	2.60	3.74	3.07			2.38
**jetski**	0%	5%	2%	Jet ski	41%	2.38	4.02	2.85			2.71
**jokercard**	0%	0%	0%	Joker Card	55%	1.71	4.71	2.45	3.72	2.84	2.48
**joustingspear**	37%	17%	2%	Spear	17%	3.31	2.31	1.84			2.25

**jumpercables**	0%	12%	5%	Jumper cables	46%	2.84	4.24	2.33			3.12
**jumpingjack**	33%	21%	2%	sander	17%	3.57	2.49	3.36			1.94
**kalashnikov**	0%	0%	0%	rifle	33%	2.43	3.86	2.48			4.06
**kangaroo01**	0%	0%	0%	kangaroo	98%	0.16	4.21	2.90	4.19	4.16	2.53
**key07**	0%	0%	0%	keys	81%	1.05	4.88	1.95	4.13	3.91	3.97
**keyboard**	0%	0%	2%	keyboard	37%	2.86	4.43	2.83			3.85
**keyhole**	0%	0%	10%	Keyhole	32%	2.19	4.64	2.19			3.41
**keyhook**	7%	10%	7%	Key hook	16%	3.60	4.25	2.22			2.64
**kidbicycle**	0%	0%	0%	Bike	33%	2.49	4.57	2.64			3.85
**kiddiepool**	0%	0%	0%	kiddie pool	26%	2.81	4.50	1.86			2.59
**kidpicnictable**	0%	0%	0%	Kids picnic Table	14%	3.88	4.12	2.24			2.24
**killerwhale01**	0%	0%	2%	Killer whale	38%	2.18	4.39	2.66			1.97
**kingfisher**	2%	7%	0%	bird	68%	1.28	3.63	3.02	3.06	3.66	1.59
**kite**	0%	0%	0%	kite	98%	0.16	4.43	2.10	2.94	3.53	3.71
**kiwi03**	0%	0%	0%	Kiwi	83%	0.89	4.71	2.38	3.72	3.47	1.91
**knee**	0%	0%	0%	Knee	88%	0.61	4.93	2.02	4.13	3.19	3.82
**kneepad01c**	21%	5%	5%	knee pad	79%	1.11	3.62	2.48	3.67	2.94	2.16
**knife03**	0%	0%	0%	knife	64%	1.50	4.81	1.67	3.84	3.56	3.85
**ladder**	0%	0%	0%	ladder	95%	0.32	4.60	1.93	3.66	4.00	4.12
**ladybug03**	0%	0%	0%	Ladybug	93%	0.48	4.69	2.79	4.53	3.44	1.53
**lamppost01**	0%	0%	2%	Lamp post	32%	2.70	4.48	2.50			2.06
**lamppost02**	0%	0%	0%	Lamp post	29%	2.71	4.43	1.83			1.76
**lantern03**	0%	2%	2%	Lantern	60%	2.11	4.02	2.45	3.44	4.19	2.74
**laundrybasket01a**	0%	0%	5%	laundry basket	50%	2.04	4.62	1.76			2.68
**lawnmower**	0%	2%	0%	lawn mower	98%	0.17	4.55	2.57	3.75	3.56	3.62
**lawnmowertractor**	0%	10%	2%	lawn mower	30%	3.13	4.05	3.10			2.79
**leafblower01**	10%	12%	0%	leaf blower	82%	1.01	3.88	2.54	3.50	3.63	2.44
**leafrake**	0%	2%	2%	rake	90%	0.55	4.79	1.57	4.44	4.00	4.15
**lectern01**	0%	7%	2%	podium	50%	2.38	4.17	2.02			2.71
**leg**	0%	0%	0%	Leg	60%	2.08	4.83	2.07	3.44	3.00	3.38
**licenseplate**	0%	0%	2%	License Plate	85%	0.80	4.83	1.54	4.13	3.44	1.97
**lifesaver**	0%	7%	26%	Life saver	32%	3.09	4.36	1.79			3.12
**lighthouse**	0%	0%	2%	Lighthouse	90%	0.54	4.10	2.71	4.06	3.72	1.91
**lightswitch01**	0%	0%	0%	Light switch	98%	0.16	4.83	1.64	4.03	4.06	4.26

**lily**	2%	5%	2%	flower	89%	0.70	3.52	3.17	2.77	3.59	1.88
**lilypad**	0%	2%	7%	Lily pad	55%	2.06	4.26	1.98			1.56
**linedpaper**	0%	0%	0%	Paper	36%	2.98	4.95	1.50			3.41
**lintroller**	0%	12%	5%	Lint roller	66%	1.83	4.36	1.71	4.11	4.06	2.97
**lion**	0%	0%	0%	lion	88%	0.61	4.40	3.02	4.03	3.31	2.18
**lionstatue**	0%	26%	0%	lion statue	61%	1.85	3.57	2.60			1.56
**lip**	0%	0%	0%	lips	86%	0.77	4.95	2.33	4.13	4.16	4.12
**litterbox**	0%	12%	2%	Litter box	28%	3.14	4.00	1.98			2.09
**lizard**	0%	2%	0%	Lizard	68%	1.33	3.79	3.02	3.75	3.81	1.82
**locker**	0%	0%	0%	locker	98%	0.16	4.76	1.60	3.97	4.47	3.12
**locomotive**	5%	2%	0%	Train	58%	2.24	3.88	3.61	3.22	2.53	2.29
**lollipop04**	0%	0%	0%	lollipop	83%	0.93	4.76	1.57	3.75	3.03	3.74
**loppingshears**	0%	10%	7%	pliers	26%	3.29	4.07	2.29			2.94
**lunchbag**	0%	2%	0%	Lunch bag	41%	2.07	4.50	2.31			2.65
**lychee01**	33%	5%	5%	Lychee	50%	2.46	2.76	2.46			1.32
**lynx01**	0%	2%	0%	Lynx	39%	2.35	4.05	3.17			1.56
**macaque01**	0%	0%	2%	monkey	68%	1.65	3.79	3.10	3.25	3.25	1.79
**macaroni01**	0%	0%	0%	Macaroni	60%	1.95	4.79	1.93	3.90	3.87	2.06
**machete**	0%	2%	0%	Machete	49%	1.14	4.00	1.76			3.09
**machinegun**	0%	0%	0%	gun	31%	2.64	3.76	2.90			4.21
**magneticcompass**	0%	2%	0%	compass	95%	0.33	4.31	2.69	3.63	3.28	2.88
**mailbox01**	0%	0%	0%	mail box	98%	0.16	4.52	2.00	2.97	3.78	2.94
**mailbox02**	0%	0%	2%	mail box	100%	0.00	4.69	2.00	4.16	3.97	3.00
**mailtruck**	0%	0%	0%	truck	31%	2.89	4.60	2.50			2.26
**makiroll**	0%	0%	0%	Sushi	81%	1.00	4.45	3.02	3.91	3.72	2.15
**manholecover**	0%	5%	7%	Sewer	19%	3.44	4.34	2.02			2.06
**maplebuttercone**	0%	7%	10%	Ice cream cone	54%	2.17	4.38	2.19			2.42
**maraca01**	24%	19%	10%	Maraca	50%	2.32	2.73	2.54			2.39
**marble**	0%	2%	2%	marble	85%	0.90	4.32	2.27	4.09	4.09	2.41
**martiniglass01b**	0%	0%	0%	martini glass	64%	1.64	4.60	1.98	4.28	2.63	3.09
**mask04**	14%	7%	0%	mask	76%	1.41	3.18	2.88	2.88	3.88	1.70
**masquerademask01**	0%	0%	0%	mask	73%	1.39	4.17	2.10	2.91	3.47	2.35
**mathcompass**	0%	12%	26%	compass	50%	2.59	3.90	2.46			2.74
**mattress**	0%	0%	0%	mattress	98%	0.16	4.86	2.05	4.06	3.69	3.26

**megaphone**	0%	8%	3%	Megaphone	42%	2.41	4.23	2.25			4.12	
**memorialplaque**	0%	21%	2%	Plaque	19%	3.82	3.90	2.26			1.68	
**menwashroomsign**	0%	2%	0%	Men’s washroom Sign	37%	3.02	4.68	1.49			2.44	
**microphone01**	0%	0%	0%	microphone	88%	0.53	4.60	2.26	4.16	3.50	4.21	
**microwave**	0%	0%	0%	microwave	81%	0.70	4.69	2.19	4.13	3.91	2.97	
**militaryhat**	10%	7%	0%	hat	43%	3.28	3.10	2.88			2.15	
**minifridge**	0%	0%	0%	Fridge	36%	2.51	4.45	1.93			2.79	
**mirror02**	0%	0%	0%	mirror	76%	1.49	4.52	2.05	2.81	3.16	3.32	
**missile**	7%	2%	2%	rocket	57%	1.55	3.24	1.79			2.18	
**mitresaw**	12%	12%	5%	Power saw	13%	3.68	3.54	3.61			2.76	
**monarchbutterfly**	0%	0%	0%	butterfly	67%	0.92	4.62	3.24	4.53	3.38	1.91	
**monument**	24%	31%	5%	obelisk	18%	3.69	2.83	2.83			1.36	
**moon**	12%	0%	0%	Moon	78%	1.35	4.37	2.51	3.94	4.23	2.27	
**moose**	0%	0%	2%	Moose	95%	0.33	4.29	3.07	4.47	4.31	1.94	
**morningstar**	31%	19%	2%	Mace	60%	2.17	2.92	2.63			1.68	
**mortarandpestle**	0%	12%	12%	Mortar and pestle	56%	2.53	4.17	2.12			3.21	
**moth**	2%	0%	0%	Moth	65%	1.44	4.07	3.34	3.59	3.65	1.50	
**motorboat02**	0%	0%	0%	boat	60%	2.04	4.17	2.86	3.22	3.66	2.50	
**motorcycle**	0%	0%	0%	motorcycle	86%	0.87	4.55	3.48	4.00	4.00	4.00	
**mouse**	0%	0%	0%	mouse	86%	0.91	4.33	2.88	3.44	3.53	2.03	
**mrpotatohead**	0%	2%	0%	Mr. Potato Head	76%	1.30	4.48	2.62	4.03	3.91	2.03	
**muffintray01**	0%	2%	0%	Muffin tray	27%	3.14	4.69	1.90			2.32	
**mug05**	0%	0%	0%	Mug	50%	1.72	4.88	1.38			3.82	
**mummy**	0%	0%	0%	mummy	86%	0.85	4.00	3.52	2.91	2.56	1.68	
**musicalwoodenspoons**	29%	26%	2%	Wooden spoons	22%	3.06	3.21	2.08			2.53	
**musicsheet**	0%	2%	2%	music sheet	80%	1.22	4.36	2.74	4.03	3.59	2.94	
**musicstand**	0%	17%	5%	Music stand	67%	1.48	4.31	2.00	4.22	3.72	2.71	
**musket**	0%	0%	2%	rifle	34%	2.85	3.79	2.26			3.82	
**muskox**	2%	7%	10%	bison	32%	2.73	3.55	3.19			1.29	
**mussel**	0%	2%	5%	Clam	38%	2.37	4.12	2.88			2.06	
**nailgun**	24%	14%	0%	Nail gun	46%	2.86	3.22	3.20			2.18	
**nametag01a**	0%	2%	12%	Name tag	33%	3.43	4.40	1.83			2.64	
**napkin**	2%	2%	7%	Napkin	41%	2.89	4.02	2.57			1.85	
**narwhal**	10%	24%	2%	Narwhal	52%	2.26	3.45	3.10			1.39	

**nightstand**	0%	5%	5%	drawer	26%	3.04	4.56	1.95			2.15
**nintendods**	0%	2%	0%	Nintendo ds	44%	2.23	4.26	2.36			3.09
**noisemaker**	2%	29%	26%	Noisemaker	28%	3.41	4.22	2.10			3.53
**noparkingsign**	0%	0%	0%	no parking sign	95%	0.32	4.74	1.69	3.94	4.03	2.06
**nose**	0%	0%	0%	nose	88%	0.53	4.95	2.31	4.09	3.44	4.03
**nosmokingsign**	0%	0%	0%	No smoking sign	90%	0.60	4.86	1.48	4.38	3.44	2.65
**nunchuk**	12%	17%	5%	Nunchuks	96%	0.22	3.60	2.03	3.91	3.75	3.44
**nyala**	5%	19%	2%	antelope	19%	3.38	3.51	3.34			1.32
**oats**	5%	8%	0%	oats	31%	2.86	3.90	2.49			1.80
**officecabinet**	0%	5%	5%	Cabinet	36%	2.69	4.33	1.68			2.50
**officechair03**	0%	0%	0%	Office chair	36%	2.31	4.67	2.26			3.47
**onewaysign**	0%	5%	0%	One way Sign	33%	3.27	4.57	1.48			2.35
**ostrich**	0%	5%	2%	Ostrich	95%	0.29	3.98	3.00	4.34	3.41	2.00
**outdoorchair**	0%	0%	0%	chair	45%	2.58	4.43	2.10			3.21
**outdoorfireplace**	12%	17%	5%	Fireplace	50%	2.52	3.38	2.67			2.09
**outdoorheater**	24%	14%	7%	outdoor heater	30%	2.99	3.13	2.71			2.06
**oven**	0%	0%	0%	Stove	45%	1.77	4.88	2.43			3.24
**owl**	0%	5%	0%	owl	95%	0.29	4.37	3.34	3.41	2.81	2.03
**oyster02**	17%	7%	5%	Oyster	53%	2.39	3.41	3.15			2.00
**paddleball**	2%	26%	14%	Paddle Ball	29%	3.12	3.90	1.90			3.56
**painting**	0%	0%	0%	Painting	71%	1.65	3.90	3.02	2.88	3.84	1.94
**palmier**	26%	10%	12%	Pastry	23%	3.62	3.72	2.60			1.54
**panda**	0%	0%	0%	panda	67%	0.92	4.55	3.02	3.97	3.03	1.85
**paninigrill**	2%	10%	0%	grill	38%	3.42	3.74	2.48			2.47
**paperairplane**	0%	0%	0%	paper airplane	69%	1.08	4.71	1.76	4.03	3.66	3.79
**paperchain**	0%	24%	10%	Paper chain	32%	3.38	3.88	1.90			1.79
**papershredder**	12%	7%	2%	paper shredder	55%	1.70	3.88	2.10			2.32
**parachute**	0%	5%	7%	parachute	95%	0.36	3.95	2.71	3.41	3.78	2.82
**parkbench02**	0%	0%	0%	bench	50%	1.54	4.71	2.40			2.94
**parkfountain**	0%	0%	0%	fountain	64%	1.07	4.43	2.60	3.34	4.00	1.79
**parkinggate**	5%	43%	12%	Parking gate	18%	3.57	4.05	2.45			2.68
**parkingmeter**	0%	2%	12%	Parking meter	83%	0.86	4.52	2.29	4.03	3.72	2.97
**parrot01**	0%	2%	0%	Parrot	71%	1.25	4.10	3.19	4.00	3.66	2.18
**patiochair**	0%	0%	0%	chair	67%	1.53	4.60	1.88	2.81	3.75	3.47

**patioumbrella**	0%	7%	12%	umbrella	53%	2.20	4.38	2.05			3.29
**pavedsidewalk**	0%	5%	5%	Bricks	18%	3.85	4.10	2.37			1.79
**payphone**	0%	0%	0%	Payphone	60%	1.97	4.71	2.76	4.31	3.97	4.21
**peacock**	0%	2%	2%	peacock	98%	0.17	4.19	3.64	3.91	2.97	2.00
**pecan02**	0%	2%	7%	Walnuts	45%	1.84	4.40	2.43			1.97
**pedestriancrossingsign**	0%	2%	2%	Pedestrian crossing Sign	18%	3.43	4.60	1.67			1.88
**penguin**	2%	5%	0%	penguin	87%	0.80	4.00	3.07	2.88	4.09	1.71
**penne**	0%	0%	2%	pasta	39%	2.57	4.81	1.81			1.97
**phonebooth**	0%	0%	0%	Phone booth	40%	2.59	4.34	2.54			3.50
**phonejack**	7%	12%	10%	Phone jack	20%	4.12	3.98	1.93			2.03
**photocopier**	0%	0%	0%	Photocopier	33%	2.78	4.52	3.10			2.94
**pickaxe02**	0%	12%	14%	Pickaxe	55%	2.43	3.98	1.79			3.29
**picnictable**	0%	0%	0%	Picnic Table	48%	2.13	4.74	2.10			2.68
**pig**	0%	0%	0%	pig	90%	0.60	4.36	2.57	3.81	3.72	2.24
**pigeon**	0%	0%	0%	Pigeon	79%	0.86	4.50	2.98	4.38	3.31	1.91
**pilgrimhat**	0%	7%	0%	hat	69%	1.79	3.57	2.31	2.78	4.03	2.74
**pillar**	26%	12%	2%	Pillar	32%	2.52	2.93	1.86			1.56
**pin**	0%	0%	7%	pin	69%	1.57	4.40	1.81	2.44	2.50	2.79
**pingpongtable**	0%	0%	0%	Ping pong Table	79%	1.13	4.57	2.12	4.28	3.78	3.68
**pintofbeer**	0%	0%	0%	glass of beer	17%	3.88	4.69	1.74			3.21
**pipe**	7%	5%	2%	pipe	50%	2.50	3.81	1.73			1.88
**pipewrench**	5%	10%	10%	wrench	47%	2.41	3.83	2.10			2.62
**pirateflag**	0%	0%	0%	Pirate flag	74%	1.27	4.17	2.10	4.25	3.03	2.21
**pitchfork**	0%	7%	7%	Pitch fork	56%	1.92	4.10	1.88			3.09
**plantpot**	0%	0%	0%	pot	26%	3.05	4.19	1.64			1.97
**platypus**	7%	7%	12%	platypus	81%	0.99	3.69	3.05	3.41	3.50	1.29
**playground**	0%	7%	2%	Playground	26%	3.36	4.26	3.02			2.32
**podium**	2%	12%	2%	podium	34%	3.09	3.85	1.68			2.47
**pokerchips**	0%	2%	2%	Poker chips	83%	0.87	4.21	2.05	4.06	3.78	3.03
**pokerset**	0%	10%	0%	Poker set	45%	2.93	4.12	2.90			2.85
**polarbear**	0%	0%	0%	Polar bear	90%	0.45	4.55	3.14	4.25	3.38	1.97
**policetape**	0%	2%	5%	police tape	54%	2.61	4.33	1.74			2.12
**poloshirt**	0%	0%	2%	shirt	32%	3.08	4.60	1.83			3.18
**poolcue**	2%	2%	5%	Pool Cue	61%	1.89	4.43	1.54	4.19	3.34	3.88

**poolnet**	0%	10%	2%	net	32%	2.78	4.21	2.43			3.15
**pooltable**	0%	0%	0%	Pool table	98%	0.17	4.66	1.98	4.16	3.47	3.76
**pooltriangle**	5%	19%	5%	pool triangle	30%	2.97	3.81	1.64			3.06
**popcorn**	2%	0%	0%	popcorn	80%	0.92	4.67	2.14	3.56	3.44	2.58
**porcupine**	0%	5%	0%	porcupine	93%	0.45	3.95	3.26	4.16	3.53	1.68
**porsche**	0%	0%	0%	Car	38%	2.64	4.36	2.88			3.47
**portapotty**	0%	0%	0%	Port-a-potty	40%	2.96	4.43	2.02			2.71
**postalmailbox01**	0%	0%	0%	mail box	83%	1.07	4.60	2.00	2.34	3.09	3.32
**postitnote**	12%	5%	7%	Post it notes	22%	3.33	4.08	1.59			2.19
**pottery**	7%	14%	0%	pottery	15%	3.93	3.50	2.45			2.09
**pouch01b**	0%	7%	2%	Pouch	29%	3.66	4.27	1.83			2.32
**powerchair**	0%	21%	17%	scooter	15%	3.90	4.24	2.85			3.03
**powerline**	0%	12%	17%	Power lines	30%	3.50	4.46	2.83			1.56
**pressurewasher**	26%	19%	5%	Pressure washer	29%	3.20	3.00	3.33			2.34
**pricesign**	0%	5%	0%	Price Sign	28%	3.12	4.64	1.48			1.65
**proboscismonkey**	21%	26%	0%	monkey	73%	1.46	2.71	3.33			1.47
**projectorscreen**	0%	7%	10%	Projector screen	40%	3.16	4.43	1.52			2.65
**pugdog**	0%	2%	0%	dog	49%	1.92	4.50	2.95			2.71
**puma**	0%	2%	2%	Cougar	28%	3.12	3.86	2.95			1.79
**pumpkin**	0%	0%	0%	Pumpkin	98%	0.16	4.71	2.29	4.06	4.13	2.18
**punchingbag**	0%	0%	5%	punching bag	83%	0.99	4.40	1.88	4.06	4.13	4.03
**punchingball**	0%	17%	7%	punching bag	59%	1.87	4.00	1.68			3.74
**puppettheatre**	0%	17%	2%	Puppet theatre	24%	3.47	3.76	2.98			2.18
**pusharoundtoy**	0%	19%	0%	Toy	29%	3.14	3.67	2.74			1.74
**puzzle**	0%	0%	0%	puzzle	48%	3.13	4.05	3.67			2.29
**raccoon**	5%	17%	2%	Raccoon	97%	0.20	3.81	3.21	3.84	3.44	1.62
**radiator**	0%	10%	5%	Radiator	44%	2.49	4.38	2.43			1.82
**railfence**	0%	10%	5%	fence	29%	3.60	4.32	1.76			2.09
**railwaycrossingsign**	0%	12%	2%	Railroad crossing	14%	4.24	4.33	2.29			2.09
**rainboot**	0%	0%	0%	rainboot	29%	2.54	4.64	1.79			3.03
**rainstick**	33%	24%	5%	Rain stick	19%	3.28	2.87	2.44			2.17
**ram**	5%	5%	12%	Ram	34%	2.25	4.00	3.07			1.53
**rawchicken**	0%	0%	0%	Chicken	33%	3.01	4.40	2.38			2.06
**record**	0%	5%	2%	record	49%	2.07	4.48	1.64			3.41

**recorder04**	0%	0%	2%	Recorder	51%	1.00	4.50	2.05			3.97
**recyclingbin**	0%	0%	0%	Recycling bin	79%	1.01	4.76	1.57	3.84	3.56	2.32
**redfox**	0%	0%	0%	Fox	88%	0.76	4.24	3.14	4.31	4.22	1.71
**redlionfish**	2%	19%	0%	fish	82%	1.15	2.86	4.02	2.34	4.03	1.41
**redonion**	0%	0%	0%	onion	57%	1.70	4.76	2.26	3.25	3.63	2.59
**revolver**	0%	0%	0%	gun	43%	2.23	4.19	2.40			4.41
**rhinoceros02**	0%	2%	5%	rhinoceros	51%	1.14	4.29	3.02			1.97
**riverotter**	7%	7%	2%	otter	66%	1.77	3.51	3.10	4.06	3.28	1.59
**road02**	0%	0%	0%	Road	71%	1.73	4.79	1.69	3.63	3.28	2.59
**rockbass**	0%	7%	0%	fish	90%	0.68	3.83	2.79	3.63	3.56	2.03
**rockingchair**	0%	0%	0%	rocking chair	55%	1.61	4.69	2.40	4.03	3.88	3.68
**rockinghorse**	2%	7%	2%	Rocking horse	32%	2.78	3.71	2.76			2.41
**rocklobster**	10%	10%	10%	Lobster	33%	2.30	3.29	3.26			1.45
**rollerblade**	0%	0%	0%	Rollerblade	83%	0.75	4.57	2.74	4.16	3.59	3.38
**rollercoaster**	2%	2%	0%	rollercoaster	48%	1.57	4.02	2.83			3.18
**rooster**	0%	2%	0%	rooster	95%	0.33	4.52	3.12	4.22	4.09	2.41
**rose**	0%	0%	0%	rose	79%	0.95	4.71	2.74	3.56	3.88	2.76
**rowboat**	5%	2%	2%	boat	50%	1.65	4.00	2.21			2.97
**rubikcube**	0%	0%	5%	Rubik cube	95%	0.34	4.68	2.83	4.48	4.26	3.91
**ruins**	0%	12%	5%	Ruins	38%	3.22	3.49	3.12			1.35
**safarihat**	0%	0%	0%	hat	40%	2.69	3.98	1.79			2.53
**safe**	0%	2%	0%	Safe	80%	1.25	4.26	2.36	3.50	2.44	3.38
**safetyglasses**	0%	0%	0%	Safety glasses	21%	2.95	4.55	2.05			3.21
**sailboat**	0%	0%	0%	Sailboat	69%	1.46	4.07	3.24	3.69	3.97	2.56
**saintbernard**	0%	0%	0%	dog	62%	1.65	4.36	2.95	3.03	3.44	2.82
**saladspinner**	7%	17%	5%	Salad spinner	37%	3.44	3.79	2.45			2.41
**salmon**	2%	2%	0%	fish	74%	1.57	3.56	2.76	3.28	3.63	2.26
**salsa**	5%	10%	5%	Salsa	35%	3.48	3.88	2.29			1.74
**saltshaker03a**	0%	0%	0%	Salt shaker	86%	0.77	4.76	1.74	4.19	3.44	3.59
**sandbagthrowgame**	45%	26%	5%	Game	20%	3.12	2.38	2.41			2.06
**sandbarshark**	0%	0%	0%	shark	90%	0.60	4.21	2.95	4.13	3.25	1.85
**sandcastle**	0%	2%	0%	Sand castle	95%	0.28	4.36	2.60	2.91	3.09	2.09
**sapsucker**	0%	12%	0%	bird	84%	0.64	3.76	3.38	3.25	2.50	1.74
**sardinecan**	2%	10%	0%	Sardine can	35%	2.88	4.12	1.95			2.41

**satellitedish01**	5%	7%	5%	Satellite dish	49%	2.14	3.67	2.81			1.71
**sausage**	0%	0%	0%	Sausage	71%	1.32	4.24	2.17	2.88	3.72	1.88
**saxophone**	0%	2%	0%	saxophone	85%	0.73	4.19	3.21	4.33	3.94	4.18
**scalpel**	2%	19%	2%	Scalpel	56%	2.28	3.40	2.07			2.47
**scanner**	0%	5%	0%	scanner	53%	2.23	4.26	2.88			3.09
**schoolbus**	0%	0%	0%	school bus	98%	0.16	4.76	2.69	4.25	3.75	2.24
**scissorlift**	10%	50%	5%	lift	33%	3.00	3.12	3.32			2.09
**scooter**	0%	5%	2%	scooter	90%	0.63	4.43	2.07	3.06	3.38	4.00
**scorpion**	0%	2%	2%	scorpion	90%	0.57	3.93	3.17	3.84	3.00	1.85
**seagull**	0%	0%	0%	Seagull	71%	1.32	4.48	2.76	3.94	2.66	1.85
**seal**	0%	0%	0%	seal	88%	0.69	4.36	2.79	3.84	3.03	1.82
**sealion**	0%	2%	0%	seal	76%	1.12	4.02	3.07	3.78	2.69	1.76
**seaturtle**	0%	0%	0%	turtle	62%	1.53	4.40	3.36	3.53	3.00	2.15
**securitycamera**	0%	2%	2%	Security camera	40%	2.50	4.19	2.40			1.79
**servingspoon**	0%	0%	0%	Spoon	50%	2.23	4.67	1.57			3.50
**shed02**	0%	5%	0%	shed	60%	2.40	4.00	2.52	3.31	3.38	2.09
**sheep**	0%	0%	0%	Sheep	93%	0.37	4.43	2.90	4.25	4.03	2.09
**shelf**	0%	2%	0%	Shelf	61%	2.03	4.40	1.71	3.50	3.88	2.44
**shellpasta**	0%	2%	2%	Shell pasta	40%	2.14	4.40	2.10			2.06
**shelves**	0%	0%	2%	Shelves	66%	1.88	4.43	1.95	2.78	3.41	2.38
**sheriffhat**	0%	0%	0%	Sheriff hat	67%	1.81	4.21	2.07	3.94	3.91	2.74
**shield02**	5%	5%	2%	Shield	73%	1.61	3.73	2.31	3.00	2.63	2.70
**shinpad**	7%	2%	7%	Shin pad	32%	2.48	4.20	1.88			2.44
**shipinabottle**	0%	10%	0%	Ship in a bottle	71%	1.51	3.98	3.21	3.81	2.97	1.97
**shoepolish**	36%	12%	7%	Shoe polish	26%	3.18	3.15	1.74			2.32
**shoppingcart**	0%	0%	2%	Shopping cart	68%	1.68	4.71	2.38	3.81	3.66	3.32
**shopvac**	0%	5%	5%	vacuum	39%	2.52	4.38	2.45			3.09
**shotglass**	24%	12%	0%	Shot glass	15%	4.09	3.18	1.83			1.93
**shoulder**	0%	0%	0%	shoulder	64%	1.94	4.93	2.48	3.72	3.72	3.29
**shoulderpad**	2%	10%	5%	Shoulder pads	31%	3.29	3.79	2.86			2.53
**shovel01**	0%	0%	2%	shovel	83%	0.94	4.71	1.52	4.00	3.94	3.97
**shower**	0%	0%	0%	shower	83%	0.94	4.57	2.69	2.75	3.34	3.56
**showerhead01**	0%	0%	2%	shower head	88%	0.72	4.79	2.29	4.03	3.81	3.53
**shrimp**	2%	12%	2%	shrimp	71%	1.39	3.67	3.14	2.44	3.63	1.68

**siamesecat**	0%	2%	0%	cat	68%	1.28	4.55	2.79	3.47	3.00	2.79
**sink**	0%	0%	0%	sink	74%	1.16	4.81	2.19	3.45	3.16	3.62
**skateboard**	0%	0%	2%	skateboard	100%	0.00	4.52	1.88	4.44	3.59	4.21
**skiboot01a**	0%	2%	2%	ski boot	70%	1.71	3.90	2.55	4.28	3.94	2.85
**skigoggles01**	0%	2%	0%	Ski goggles	44%	1.83	4.26	1.83			3.09
**skihelmet01**	0%	0%	0%	helmet	64%	1.64	4.14	1.95	2.75	3.28	3.18
**skipole**	0%	10%	5%	Ski poles	67%	1.79	4.33	1.81	4.22	3.97	3.29
**skippingrope**	0%	0%	0%	skipping rope	52%	1.13	4.55	1.60	3.44	3.31	4.26
**skunk**	0%	2%	0%	skunk	98%	0.17	4.33	2.83	4.41	3.56	2.35
**sledgehammer**	0%	0%	0%	hammer	60%	1.31	4.26	1.83	2.87	2.71	3.59
**slug**	5%	2%	2%	slug	68%	1.16	3.85	2.46	3.97	3.81	1.68
**slushiemachine**	0%	0%	10%	Slushie Machine	58%	2.51	4.24	2.74	3.63	3.66	2.33
**smartboard**	5%	0%	0%	white board	60%	2.28	4.37	1.62	4.53	4.19	3.03
**smokedetector02**	5%	2%	5%	Smoke detector	54%	1.76	4.48	2.02			2.12
**smokedsalmon**	0%	0%	0%	Salmon	29%	3.22	4.14	2.57			1.64
**smokingpipe**	0%	0%	2%	pipe	83%	0.76	4.10	1.79	3.91	3.53	3.65
**snail**	0%	0%	5%	shell	40%	2.37	4.02	2.71			1.79
**snowblower**	7%	19%	5%	snow blower	41%	2.77	3.55	3.52			2.35
**snowboard**	0%	0%	0%	Snowboard	95%	0.32	4.50	2.29	4.03	2.94	3.65
**snowglobe**	0%	10%	0%	snow globe	68%	1.87	4.12	2.90	3.34	4.00	2.15
**snowman**	0%	0%	0%	snowman	100%	0.00	4.62	2.26	4.09	4.22	3.26
**snowshoe**	0%	17%	5%	Snowshoe	88%	0.63	3.57	2.71	3.97	3.44	3.12
**snowshovel**	0%	0%	2%	Snow shovel	56%	0.99	4.62	1.69	4.06	4.13	4.03
**soccerball**	0%	0%	0%	soccer ball	98%	0.16	4.52	1.98	3.84	4.22	3.97
**soccercleat01**	0%	0%	0%	Soccer cleat	26%	2.59	4.38	2.43			3.18
**soundmixer**	12%	12%	7%	Sound mixer	17%	3.82	3.31	3.74			2.88
**spacerocket**	2%	0%	2%	rocket	68%	1.77	3.93	2.45	3.44	4.31	2.03
**spacerover**	31%	10%	0%	Mars rover	56%	2.16	2.53	3.73			1.50
**spaghetti01**	0%	0%	0%	Spaghetti	57%	2.35	4.76	1.79	3.06	3.44	2.50
**spear02**	5%	2%	7%	Spear	75%	1.28	3.71	1.63	4.13	3.63	2.97
**speedball**	0%	24%	5%	punching bag	50%	2.30	3.78	2.37			3.82
**spicerack**	2%	2%	0%	spice rack	85%	0.88	4.36	2.93	3.00	3.53	2.74
**spiderweb**	0%	0%	0%	spider web	93%	0.44	4.74	2.71	4.38	4.06	2.38
**spinningtoy**	5%	10%	0%	Toy	36%	2.57	3.38	2.88			1.64

**sportsjersey**	0%	0%	0%	jersey	52%	2.43	4.36	1.86	2.39	2.61	2.32
**spotlight**	0%	7%	0%	spot light	38%	2.67	3.83	2.38			2.28
**springdoorstop**	5%	21%	7%	Door stop	43%	1.91	4.40	2.14			2.00
**springroll**	0%	0%	2%	Spring roll	54%	1.87	4.59	1.86	4.06	3.75	1.85
**sprinkler**	2%	14%	10%	Sprinkler	39%	3.04	4.00	2.69			2.26
**sprouts**	12%	21%	7%	Sprouts	28%	3.43	3.55	2.17			1.66
**squid**	7%	5%	5%	Squid	88%	0.70	3.65	3.38	3.22	3.16	1.58
**squirrel**	0%	0%	0%	squirrel	100%	0.00	4.69	2.88	3.72	3.38	1.94
**stairs**	0%	0%	0%	stairs	62%	1.58	4.69	2.10	2.91	3.50	3.59
**staplegun**	5%	10%	2%	Staple gun	40%	2.17	4.24	2.26			3.29
**star**	10%	5%	0%	Star	58%	2.23	3.24	2.50			1.71
**statue**	0%	2%	0%	statue	88%	0.82	3.67	2.90	3.13	3.97	1.64
**steamroller**	0%	21%	19%	Steam roller	52%	2.37	4.05	3.10			2.18
**steeringwheel**	0%	0%	0%	Steering wheel	68%	1.36	4.76	2.15	4.13	2.91	4.32
**stepladder**	0%	0%	0%	ladder	83%	0.75	4.62	1.76	3.61	3.61	3.47
**stingray**	0%	14%	7%	Stingray	52%	2.17	3.48	3.19			1.47
**stool01**	0%	0%	0%	Stool	88%	0.76	4.67	1.81	4.16	3.00	3.12
**storagebin**	0%	17%	5%	Shelves	12%	4.15	3.90	1.76			1.88
**stovetop**	0%	2%	5%	Stove top	18%	3.78	4.59	2.05			2.68
**strap**	2%	21%	12%	Belt	56%	2.51	3.86	1.71			2.52
**stroller**	0%	2%	5%	Stroller	56%	1.49	4.43	2.79	4.00	3.88	3.18
**stubbywrench01a**	0%	5%	12%	wrench	100%	0.00	4.41	1.66	4.13	3.59	3.32
**stuffedpuffin**	7%	14%	0%	bird	61%	1.56	3.24	3.39			1.56
**sturgeon**	12%	17%	0%	fish	79%	1.13	2.59	3.20	2.41	3.00	1.53
**suitofarmor**	0%	0%	5%	armor	53%	2.18	3.98	3.17			2.18
**sunflowerseeds**	2%	0%	2%	Sunflower seeds	50%	2.17	4.40	2.45			2.00
**surfboard**	12%	2%	5%	surf board	100%	0.00	3.61	1.85	3.78	3.09	3.38
**swallow**	0%	2%	0%	bird	71%	1.64	3.69	3.10	3.34	2.94	1.79
**swan**	0%	5%	0%	bird	38%	2.09	3.93	2.79			1.88
**swimsuit**	0%	0%	0%	Shorts	55%	2.40	4.67	2.10	3.42	2.69	2.79
**swing**	0%	0%	0%	swing	98%	0.17	4.61	1.59	3.13	3.50	4.00
**swissarmyknife01a**	0%	2%	2%	pocket knife	43%	2.34	4.36	2.05			2.97
**sword01**	0%	0%	0%	sword	81%	1.08	4.07	2.02	4.06	3.58	3.62
**swordfish**	0%	2%	2%	sword fish	83%	0.67	4.05	2.90	4.09	3.88	1.62

**tabascosauce**	2%	2%	2%	Tabasco Sauce	38%	2.86	4.17	2.05			2.71
**table01**	0%	0%	0%	table	81%	1.08	4.79	1.71	3.19	3.94	2.88
**tablesaw**	29%	17%	5%	Table saw	67%	1.85	3.03	3.02			2.18
**taillight**	2%	7%	2%	Tail lights	27%	3.02	4.21	2.52			1.74
**tambourine03**	0%	7%	5%	Tambourine	65%	1.46	4.19	2.36	3.68	3.50	3.88
**tank**	0%	5%	10%	Tank	75%	1.17	4.31	3.24	3.66	3.25	2.06
**tankertruck**	0%	2%	0%	truck	49%	2.84	4.45	2.95			2.03
**tarantula**	0%	0%	0%	Tarantula	67%	1.16	4.10	3.45	3.78	3.38	1.94
**taxisign**	0%	7%	2%	taxi sign	68%	1.67	4.40	1.62	3.19	2.94	2.74
**telescope**	0%	2%	2%	telescope	95%	0.34	4.14	2.40	3.19	3.75	3.59
**television**	0%	0%	0%	TV	24%	3.48	4.76	1.78			3.56
**tennisracket**	0%	0%	0%	Tennis racket	62%	1.29	4.36	2.19	3.88	3.41	4.03
**tent**	0%	0%	0%	tent	90%	0.60	4.45	2.14	3.09	3.84	2.82
**tern**	5%	12%	2%	bird	88%	0.70	3.26	3.07	3.16	3.63	1.85
**tie02**	0%	0%	0%	tie	79%	0.75	4.64	1.52	3.75	4.38	4.21
**tiger02**	0%	0%	0%	tiger	95%	0.32	4.36	3.12	3.97	3.97	1.82
**tire**	0%	0%	2%	tire	78%	1.14	4.62	1.60	4.16	3.88	2.56
**toad**	0%	0%	0%	frog	55%	1.13	4.12	3.62	3.22	2.72	2.15
**toasteroven**	0%	0%	0%	Toaster oven	67%	1.85	4.43	3.00	3.88	3.66	2.79
**toilet**	0%	0%	0%	Toilet	90%	0.53	4.93	2.24	3.84	3.72	4.53
**toiletbrush**	0%	5%	10%	Toilet brush	67%	1.78	4.57	2.12	4.09	3.06	3.12
**tombstone**	0%	0%	0%	Tombstone	69%	1.56	4.43	1.98	3.56	3.22	2.29
**tonfa**	14%	19%	2%	Baton	44%	2.84	3.45	1.71			2.27
**toolbox02**	0%	2%	2%	Toolbox	93%	0.45	4.36	2.07	3.41	3.75	2.47
**tortillachip**	0%	0%	0%	tortilla chip	38%	2.77	4.71	2.07			2.50
**tortoise01**	0%	0%	0%	turtle	76%	0.90	4.26	3.26	3.88	4.09	2.26
**totempole**	0%	5%	5%	Totem Pole	74%	1.14	4.12	3.29	3.94	3.81	1.41
**toucan**	0%	12%	10%	Toucan	76%	1.26	3.98	3.17	4.13	3.78	1.62
**towelrack**	0%	12%	2%	Towel rack	47%	2.75	4.48	1.64			2.56
**tower**	5%	2%	2%	Tower	58%	2.49	3.71	2.76	3.31	3.78	1.68
**townsquarepostclock**	0%	5%	0%	clock	45%	2.20	3.83	2.52			2.03
**toyanimal05**	2%	2%	0%	toy horse	80%	1.12	3.95	2.05	2.38	3.34	1.74
**toyfiretruck**	0%	5%	0%	toy truck	53%	2.07	4.36	1.95			2.12
**toynutcracker**	0%	10%	0%	nutcracker	66%	1.76	3.83	2.88	3.34	2.91	1.88

**toysoldier01b**	0%	5%	2%	Toy soldier	72%	1.78	4.31	2.12	3.78	2.78	2.15
**toytanktruck**	0%	0%	0%	toy truck	67%	1.88	4.33	1.98	3.06	2.91	2.00
**tractor**	0%	5%	7%	tractor	38%	2.80	3.98	3.38			2.35
**trafficlight**	0%	2%	0%	Traffic light	56%	1.61	4.74	2.10	3.16	3.13	2.65
**trailer**	2%	12%	5%	Trailer	62%	2.20	3.80	2.32			1.79
**trailerhitchball**	31%	24%	0%	Trailer hitch	47%	2.64	3.34	2.13			2.17
**trampoline**	0%	0%	0%	trampoline	93%	0.37	4.40	1.98	4.03	4.20	4.06
**travelmug**	0%	0%	0%	Coffee mug	29%	3.09	4.71	1.67			3.24
**treadmill**	0%	0%	2%	Treadmill	90%	0.61	4.67	2.62	4.03	3.50	3.59
**tree**	0%	0%	0%	tree	95%	0.32	4.69	2.93	3.57	4.23	2.62
**treestump**	0%	2%	0%	Tree stump	59%	1.86	4.36	2.74	3.59	4.19	2.09
**treetrunk**	5%	0%	0%	Tree trunk	38%	2.86	4.38	2.57			1.76
**triangle**	2%	12%	7%	triangle	79%	1.22	4.05	1.45	3.13	3.47	3.41
**triangleruler01**	0%	12%	12%	Ruler	23%	3.43	4.37	1.63			2.59
**tricornhat**	10%	7%	0%	hat	57%	2.10	2.98	2.13			2.33
**tricycle**	0%	0%	2%	Tricycle	93%	0.49	4.57	2.50	3.90	3.00	2.82
**trident01**	0%	12%	2%	Trident	44%	2.47	4.00	1.67			2.24
**trojanhorse**	0%	14%	0%	Trojan horse	64%	1.68	3.69	2.95	3.47	3.20	1.44
**trombone**	0%	5%	5%	Trombone	63%	1.73	3.93	2.64	4.03	3.37	4.47
**trophy01**	0%	0%	0%	trophy	93%	0.37	4.33	2.10	3.90	4.03	2.71
**trumpet**	0%	2%	0%	trumpet	83%	0.97	4.38	2.52	4.20	4.17	4.18
**tuba**	0%	2%	2%	Tuba	35%	2.47	3.86	3.14			3.79
**tulip02**	0%	0%	0%	tulip	76%	1.21	4.40	2.12	4.23	4.17	2.35
**turkey**	0%	7%	0%	turkey	62%	1.61	3.90	3.14	2.97	3.47	1.71
**turnip**	0%	7%	0%	Turnip	41%	1.83	4.14	2.36			1.59
**turnstile**	2%	21%	12%	Turnstile	81%	1.12	4.45	2.22	3.78	3.17	3.09
**uprightpiano01**	0%	0%	0%	Piano	88%	0.69	4.67	2.98	3.73	3.87	4.47
**urinal**	7%	2%	5%	urinal	89%	0.67	4.10	1.95	3.20	2.40	3.50
**usbcable01a**	0%	5%	2%	Usb cable	44%	3.02	4.55	1.90			2.50
**vacuumcleaner01**	0%	0%	0%	vacuum	62%	1.21	4.50	2.55	3.20	3.70	3.79
**videotape01b**	7%	2%	2%	cassette	35%	2.82	3.73	2.29			1.79
**visor**	0%	5%	0%	visor	48%	2.42	4.36	1.74			3.12
**volleyball**	0%	0%	0%	Volleyball	76%	1.07	4.55	1.76	3.67	4.20	3.65
**vulture**	2%	5%	5%	Vulture	70%	1.01	3.98	3.41	3.90	3.10	1.65

**waders**	0%	21%	5%	Waders	6%	4.70	3.66	2.38			2.32
**wafflemaker**	0%	2%	0%	waffle maker	54%	2.01	4.19	2.90	3.53	3.60	2.24
**wagonwheel**	0%	0%	0%	wheel	57%	1.79	4.19	2.05	2.60	3.27	2.24
**walkietalkie**	2%	0%	2%	walkie talkie	73%	1.60	3.95	2.79	3.33	3.47	3.38
**wallclock**	0%	0%	0%	clock	88%	0.76	4.67	1.90	3.10	3.73	3.12
**walldeco01**	7%	7%	0%	Branches	22%	3.79	3.78	2.65			1.35
**warthog**	2%	14%	2%	Warthog	38%	2.15	3.45	3.17			1.35
**washingmachine**	0%	0%	2%	washing machine	63%	1.95	4.40	2.55	3.37	3.83	2.50
**waterbottleholder**	29%	17%	2%	Water bottle holder	32%	3.29	3.28	2.15			2.04
**watercolorpaintset**	0%	2%	0%	Paint	28%	3.48	4.49	2.07			3.12
**watercooler**	0%	2%	2%	Water cooler	58%	2.07	4.57	2.14	4.56	4.56	3.18
**waterfall**	0%	0%	0%	Waterfall	95%	0.28	4.14	3.00	3.17	3.43	1.76
**waterfountain02**	0%	0%	5%	water fountain	78%	1.04	4.81	2.36	3.77	4.30	3.65
**waterheater**	7%	7%	2%	Water heater	41%	2.59	3.88	2.24			1.58
**waterlily**	0%	5%	0%	flower	65%	1.79	3.95	3.05	2.73	3.60	1.91
**watervalve**	0%	17%	21%	Water valve	15%	3.67	4.37	2.39			2.97
**weathervane**	0%	20%	32%	Weathervane	35%	2.76	3.76	2.68			1.82
**webcam**	7%	2%	0%	Webcam	68%	1.60	4.17	2.43	4.00	4.37	2.09
**weddingcake**	0%	2%	0%	wedding cake	59%	1.50	4.48	2.52	3.30	3.23	2.09
**weedwacker**	12%	15%	5%	Weed whacker	32%	3.14	3.68	2.45			2.88
**well**	0%	2%	2%	Well	75%	1.15	4.19	2.74	3.00	3.87	2.91
**wetfloorsign**	0%	7%	0%	Wet floor Sign	18%	3.98	4.55	1.64			2.68
**whaleshark**	5%	17%	0%	fish	42%	1.99	3.50	3.05			1.62
**wheat**	2%	0%	12%	Wheat	72%	1.59	3.93	2.33	3.93	3.63	1.38
**wheel04**	0%	5%	2%	wheel	51%	2.41	4.33	2.05			2.47
**wheelbarrow01**	0%	10%	10%	wheel barrow	91%	0.57	4.41	2.27	3.37	3.50	3.62
**wheelchair**	0%	0%	0%	Wheelchair	98%	0.16	4.52	2.79	3.60	4.00	3.85
**whiteout**	0%	2%	0%	Whiteout	66%	1.18	4.60	2.17	3.57	2.77	2.88
**whitetiger**	0%	2%	0%	tiger	44%	2.02	4.19	3.24			1.76
**wiicontroller**	5%	2%	0%	Wii controller	26%	3.81	4.07	2.29			3.03
**windmill**	0%	5%	2%	windmill	100%	0.00	4.33	2.81	3.80	3.90	2.00
**windowblinds02**	0%	2%	0%	Blinds	46%	2.29	4.69	2.05			3.53
**windowshutter**	2%	7%	5%	window	36%	2.49	4.02	2.14			2.24
**windshieldwasherfluid**	0%	12%	10%	Windshield washer fluid	22%	3.75	4.07	1.74			1.91

**windshieldwiper02**	7%	2%	0%	Windshield Wiper	82%	1.21	4.32	2.12	4.37	3.33	3.03
**windsurfboard**	0%	17%	7%	Wind surfboard	25%	3.15	3.76	2.56			3.29
**windturbine**	0%	12%	12%	windmill	56%	1.55	4.00	1.78			1.97
**winniethepooh**	0%	0%	0%	Winnie the pooh	62%	1.97	4.52	2.45	3.33	3.97	1.56
**witchhat**	0%	0%	0%	Witch hat	86%	0.68	3.98	2.00	3.30	4.30	2.76
**womenwashroomsign**	0%	0%	0%	women's bathroom sign	29%	3.33	4.76	1.60			2.35
**woodencrate01**	10%	5%	0%	box	36%	2.46	3.61	1.71			1.88
**woodenfence**	0%	2%	5%	fence	62%	2.04	4.17	1.76	3.35	3.90	1.85
**woodenshoe**	24%	5%	0%	wooden shoe	50%	1.97	3.03	2.07			2.19
**worldmap**	0%	0%	0%	World map	67%	1.40	4.76	3.12	4.30	4.43	2.09
**xylophone**	0%	12%	2%	xylophone	89%	0.73	4.07	2.69	3.63	3.87	3.47
**zebra**	0%	0%	0%	zebra	98%	0.16	4.40	3.05	4.23	3.97	1.47
**zipper**	0%	0%	0%	zipper	100%	0.00	4.83	2.14	3.87	3.07	4.24

Nb = Number of stimuli, NA = Name Agreement, Fam = Familiarity, VC = Visual Complexity, CA = Category Agreement, OA = Object Agreement, VA = Viewpoint Agreement, Manip = Manipulability.
