# Supplementary material for: Bank of Standardized Stimuli (BOSS) Phase II: 930 New Normative Photos
Source: PLoS One. 2014 Sep 11;9(9):e106953. doi: 10.1371/journal.pone.0106953 (PMC4161371; doi:10.1371/journal.pone.0106953)
Supplement: Table S2 — List of all stimulus-specific norms of category agreement and Hcat. (PDF) [file pone.0106953.s002.pdf]

Filename	CA	Hcat
**Animal**		
**Bird**		
americangoldfinch *	97%	0.20
americanwhitepelican *	97%	0.20
barnowl *	100%	0.00
bluejay *	97%	0.20
canadiangoose *	97%	0.20
cardinal *	94%	0.39
cedarwaxwing *	100%	0.00
cormorant *	97%	0.20
duck01 *	100%	0.00
eagle *	100%	0.00
falcon *	100%	0.00
flamingo *	97%	0.20
greathornowl *	94%	0.39
hen *	97%	0.20
heron01 *	100%	0.00
kingfisher *	97%	0.20
ostrich *	100%	0.00
owl *	91%	0.60
parrot01 *	100%	0.00
peacock *	97%	0.20
penguin *	76%	1.03
pigeon *	100%	0.00
rooster *	97%	0.20
sapsucker *	97%	0.20
seagull *	97%	0.20
stuffedpuffin *	91%	0.58
swallow *	100%	0.00
swan *	100%	0.00
tern *	100%	0.00
toucan *	100%	0.00
turkey *	100%	0.00
vulture *	97%	0.20
**Canine**		
bassethound *	88%	0.63
dalmatian *	91%	0.44
fennec *	73%	0.85
greyhound *	85%	0.82
greywolf *	76%	0.80
hyena *	48%	1.35
pugdog *	91%	0.53
saintbernard *	94%	0.33

**Crustacean**		
crab01 *	94%	0.39
horseshoecrab *	55%	1.66
mussel	58%	1.96
rocklobster *	82%	0.91
seashell01	41%	1.93
shrimp *	79%	0.99
snail *	64%	1.64
starfish01 *	64%	1.64
**Feline**		
cat *	94%	0.40
cheetah *	69%	1.26
cougar *	79%	1.04
jaguar *	82%	0.80
lion *	64%	1.10
lynx01 *	87%	0.66
puma *	82%	0.80
siamesecat *	97%	0.20
tiger02 *	67%	1.28
whitetiger *	67%	1.48
**Fish**		
clownfish *	100%	0.00
cuttlefish *	45%	2.07
fishskeleton	70%	1.48
greatwhiteshark *	58%	1.83
hammerheadshark *	64%	1.32
holdfast *	97%	0.20
jellyfish *	55%	1.74
redlionfish *	91%	0.52
rockbass *	97%	0.20
salmon *	94%	0.39
sandbarshark *	73%	1.18
squid *	48%	1.75
stingray *	72%	1.26
sturgeon *	91%	0.53
swordfish *	94%	0.39
whaleshark *	64%	1.24
**Insect**		
ant *	100%	0.00
butterfly *	97%	0.20
cockroach02 *	100%	0.00
daddylonglegs *	100%	0.00
dragonfly01 *	97%	0.20
grasshopper *	97%	0.20

honeybee *	94%	0.39
ladybug03 *	97%	0.20
monarchbutterfly *	94%	0.33
moth *	91%	0.58
scorpion *	70%	1.10
slug *	64%	1.59
tarantula *	94%	0.39
**Mammal**		
africanelephant *	100%	0.00
antelope *	94%	0.40
armadillo *	58%	1.14
asianelephant *	97%	0.20
badger *	100%	0.00
bat *	79%	1.08
beaver *	94%	0.34
bison *	97%	0.20
blackbear *	97%	0.20
bull *	100%	0.00
caribou02 *	100%	0.00
chimpanzee *	94%	0.33
chipmunk *	97%	0.20
cow *	100%	0.00
dromedary *	100%	0.00
giraffe *	97%	0.20
gorilla *	94%	0.39
grizzly *	100%	0.00
hippopotamus *	94%	0.39
horse *	100%	0.00
jackrabbit *	97%	0.20
kangaroo01 *	94%	0.39
macaque01 *	94%	0.39
moose *	91%	0.60
mouse *	97%	0.20
muskox *	100%	0.00
nyala *	88%	0.71
panda *	100%	0.00
pig *	94%	0.40
platypus *	61%	1.27
polarbear *	100%	0.00
porcupine *	88%	0.71
proboscismonkey *	100%	0.00
raccoon *	97%	0.20
ram *	100%	0.00
redfox *	73%	0.85
rhinoceros02 *	97%	0.20
sheep *	100%	0.00

skunk *	91%	0.58
squirrel *	97%	0.20
warthog *	100%	0.00
zebra *	97%	0.20
**Reptile**		
alligator *	97%	0.20
chameleon *	97%	0.20
cobra *	100%	0.00
crocodile *	97%	0.20
gecko *	97%	0.20
lizard *	97%	0.20
seaturtle *	75%	1.25
toad *	100%	0.00
tortoise01 *	85%	0.72
**Sea mammal**		
belugawhale *	79%	1.04
dolphin01 *	76%	1.11
killerwhale01 *	85%	0.76
narwhal *	75%	1.14
riverotter *	52%	1.16
seal *	88%	0.53
sealion *	82%	0.68
**Bodypart**		
arm *	97%	0.20
brainmodel	59%	1.44
dinosaurskull	27%	2.29
ear *	97%	0.20
elbow *	91%	0.45
eye *	100%	0.00
fingernail02 *	73%	1.32
fingerprint	58%	2.17
foot *	97%	0.20
hand01b *	97%	0.20
humanskeleton	73%	1.36
humanskull	67%	1.54
knee *	100%	0.00
leg *	100%	0.00
lip *	100%	0.00
mummy	45%	2.48
nose *	100%	0.00
shoulder *	97%	0.20
**Building infrastructure**		
addressplate	31%	2.11

airvent	41%	1.79
awning	84%	0.93
balcony02	97%	0.20
barbedwire01	63%	1.43
barn	97%	0.20
baseboardheater01	28%	2.62
belltower01	100%	0.00
bikerack	69%	1.45
birdhouse	59%	1.74
bleachers	74%	1.22
bridge	97%	0.21
buoy	77%	1.19
busshelter	97%	0.20
castle	100%	0.00
castletower	97%	0.20
ceilingfan01	22%	2.56
chainlinkfence	69%	1.04
chimney	85%	0.72
church	97%	0.20
codedoorlock	39%	2.19
column	100%	0.00
communitymailbox	69%	1.56
compostbin	68%	1.76
crowdcontrolstanchion	39%	2.49
doghouse	82%	1.03
donotentersign	44%	2.17
donotwalksign	52%	2.01
dormer	91%	0.45
doubledoors	58%	1.53
dumpster	69%	1.45
eiffeltower	97%	0.20
electricitymeter	59%	1.65
electrictransformer	56%	1.62
entrancegate	90%	0.46
escalator	88%	0.64
exitsign	53%	2.22
fence02	82%	0.80
firealarm	31%	2.78
firehydrant	66%	1.78
fireplace	70%	1.30
fusebox	56%	1.66
gazebo	100%	0.00
gutteranddrainpipe	67%	1.21
handrail	76%	1.06
hangar	97%	0.20
icemaker	56%	2.02
lamppost01	59%	1.80

lamppost02	66%	1.66
lighthouse	97%	0.20
lionstatue	61%	1.36
locker	39%	1.93
mailbox01	47%	2.07
mailbox02	79%	1.14
manholecover	75%	1.35
memorialplaque	50%	1.56
menwashroomsign	21%	2.90
monument	85%	0.76
noparkingsign	52%	2.11
nosmokingsign	42%	2.23
officecabinet	42%	2.06
onewaysign	42%	2.58
outdoorfireplace	39%	2.26
parkfountain	94%	0.39
parkinggate	81%	1.03
parkingmeter	59%	1.97
pavedsidewalk	69%	1.04
pedestriancrossingsign	52%	2.02
phonebooth	70%	1.16
pillar	74%	1.32
portapotty	65%	1.60
postalmailbox01	63%	1.94
powerline	81%	0.93
radiator	59%	1.58
railfence	67%	1.42
railwaycrossingsign	69%	1.54
road02	64%	1.69
rollercoaster	45%	1.76
ruins	97%	0.20
sandcastle	41%	2.04
shed02	85%	0.90
sprinkler	38%	2.28
stairs	90%	0.46
tombstone	48%	2.24
tower	97%	0.20
townsquarepostclock	75%	1.14
trafficlight	52%	2.04
turnstile	88%	0.71
waterfountain02	64%	1.90
waterheater	42%	2.11
well	84%	0.93
windmill	97%	0.20
windowshutter	48%	2.12
windturbine	69%	1.64
womenwashroomsign	27%	2.39

woodenfence	78%	1.01
**Building material**		
baseboard	81%	0.82
bolt01a	50%	1.43
boltandnut02	64%	1.24
brasslipstrike	48%	1.83
brick	88%	0.63
chain01	33%	2.85
cinderblock	82%	0.80
coaster	42%	2.01
doorbolt	55%	2.05
doorhandle	53%	1.86
doorlock	45%	1.95
floortile01	66%	1.35
floortile02	70%	1.16
fluorescenttubelight01	34%	2.37
hinge	67%	1.51
insulation	85%	0.97
keyhole	53%	1.68
lbracket01	66%	1.44
nail	48%	1.33
paintcan01	44%	1.77
pipe	66%	1.69
plastictube	42%	2.53
screw02	45%	1.69
springdoorstop	52%	1.73
trailerhitchball	27%	2.59
watervalve	41%	2.18
woodboard	88%	0.78
**Clothing**		
apron	67%	1.06
athleticsock	97%	0.20
belt02a	91%	0.52
beret01	67%	1.66
bib	67%	1.59
boot02b	100%	0.00
bowtie	91%	0.60
button01	82%	1.01
cap01a	97%	0.20
cap03	91%	0.53
chefshat	78%	1.06
colonialhat	84%	0.84
crosscountryboot	52%	1.00
diaper01c	48%	1.51
eyepatch	50%	1.98

flipflop01b	94%	0.39
glasses01a	69%	1.47
glassescase	45%	2.25
graduationcap	82%	0.85
hairband	84%	0.93
handbag02a	52%	1.86
hardhat02	63%	1.68
hat03b	100%	0.00
highheelshoe01	94%	0.40
hikingshoe01	88%	0.54
jeans01	97%	0.20
manshoe	91%	0.58
militaryhat	67%	1.06
mitten04	100%	0.00
moccasin	94%	0.39
overalls	97%	0.20
pants	97%	0.20
pilgrimhat	94%	0.39
poloshirt	91%	0.58
rainboot	91%	0.44
ridinghelmet	82%	0.68
safarihat	91%	0.44
safetyglasses	36%	2.01
sandal	97%	0.20
scarf	97%	0.20
sheriffhat	94%	0.40
shirt01	100%	0.00
shirt02	100%	0.00
shoelace	88%	0.73
shorts01	100%	0.00
slipper01b	97%	0.20
sneaker03b	91%	0.44
soccercleat01	55%	0.99
sock01a	100%	0.00
sportsjersey	91%	0.44
strap	52%	1.99
sunglasses04	70%	1.16
swimsuit	94%	0.39
tie02	100%	0.00
tricornhat	85%	0.90
tshirt	100%	0.00
tuque03a	97%	0.20
visor	94%	0.33
waders	88%	0.63
wallet02a	34%	2.57
witchhat	88%	0.78
woodenshoe	76%	1.06

workglove02c	59%	1.29
zipper	94%	0.39
**Decoration & gift accessory**		
amphora	72%	1.35
angelstatue	76%	1.17
antlers	50%	1.75
aquarium	42%	2.05
balloon01b	61%	1.36
birthdaycandle	78%	1.07
box01a	85%	0.97
bracelet04	33%	1.95
brooch	55%	1.15
buddhastatue	97%	0.20
buffaloskull	34%	2.24
bust	76%	1.06
candelabra	69%	1.30
candle01	58%	1.47
candle08b	79%	0.99
candleholder01	58%	1.38
christmasball	100%	0.00
christmaslights	91%	0.44
christmastree	91%	0.52
christmaswreath	100%	0.00
clock04	67%	1.42
coathook	31%	1.98
cork01	42%	1.91
cross01	50%	1.75
curtain	42%	1.77
discoball	76%	0.80
dreamcatcher	91%	0.58
flag	33%	2.29
flowerwreath	97%	0.20
gardengnome02	94%	0.33
garland	82%	0.80
gift01	88%	0.71
giftbag01a	85%	0.90
giftbow02b	100%	0.00
giftbow04	97%	0.20
giraffefigurine	85%	0.82
handfan01b	84%	0.93
horsefigurine	85%	0.76
jackolantern	64%	1.24
Jesusstatue	52%	1.79
jewelrybox01b	73%	1.23
lantern03	47%	2.34
lei	61%	1.71

mask02a	97%	0.20
mask04	84%	0.86
masquerademask01	59%	1.34
medal02b	47%	2.12
mirror01	77%	1.31
napkin	56%	1.92
noisemaker	52%	1.16
painting	94%	0.33
paperchain	72%	1.20
partyhat	73%	1.24
pictureframe04	79%	0.99
piggybank	48%	2.01
pin	55%	2.00
policetape	24%	2.75
pottery	58%	2.07
pouch01b	72%	1.44
pricesign	24%	2.70
ribbon03a	82%	0.80
ribbon04	97%	0.20
shipinabottle	84%	0.74
slide02	64%	1.46
snowglobe	88%	0.64
star	55%	2.04
statue	45%	1.49
statuette01	88%	0.73
strawbasket01	85%	0.82
totempole	81%	1.03
toynutcracker	52%	1.16
trojanhorse	36%	2.35
trophy01	59%	1.74
vase01	75%	0.95
vase02	79%	0.95
vase03	81%	0.93
walldeco01	88%	0.71
weathervane	31%	2.26
windowblinds02	27%	2.30
winniethepooh	70%	1.10
**Electronic device & accessory**		
airconditioner	63%	1.62
alarmclock01	97%	0.20
alarmsystem	72%	1.48
antenna	88%	0.78
atm	58%	1.49
audiocablesplitter	94%	0.40
avcable	91%	0.58
battery02b	64%	1.46

camera01a	79%	1.20
cameracase01a	48%	2.17
carantenna	24%	2.67
carbattery	31%	2.26
carlighter02	36%	2.52
cashregister01	82%	1.03
cassettetape01a	79%	0.95
cd	88%	0.64
ceilingspeaker	52%	2.45
cellphone	97%	0.20
chandelier	28%	2.53
clock01a	30%	2.11
coffeemachine	39%	1.84
computerkeyboard02	100%	0.00
computermouse06	97%	0.20
crocodileclips	84%	0.93
desktopcomputer	100%	0.00
djmixer01	48%	1.53
djmixer02	45%	1.43
dvdcase01	58%	1.67
dvdplayer	84%	0.78
electricoutlet	45%	1.99
ethernetcable02	88%	0.78
expansioncard	94%	0.39
extensioncord	88%	0.73
fan	44%	2.17
fanblades	27%	3.03
fanheater	67%	1.48
faxmachine	94%	0.33
filmnegative	39%	1.98
filmroll	64%	1.76
floppydisk02a	100%	0.00
fluorescentlightbulb	41%	2.00
fuse	30%	2.70
gps	82%	0.87
halogenlightbulb	41%	2.10
headphones02b	76%	1.14
heatpump	31%	2.50
ipad02	94%	0.39
ipod	94%	0.39
jack	45%	2.35
lamp04a	36%	2.25
laptop01a	100%	0.00
lectern01	56%	1.31
lightbulb01	44%	1.86
lightswitch01	39%	1.99
megaphone	48%	1.78

microphone01	59%	1.52
monitor	94%	0.39
nintendods	73%	1.05
outletadapter01	50%	2.07
papershredder	41%	1.94
payphone	88%	0.64
pda	85%	0.76
phonejack	58%	1.61
photocopier	73%	0.85
powercable	100%	0.00
printer02	85%	0.61
radio01	70%	1.24
remotecontrol04	91%	0.60
satellitedish01	84%	0.75
scanner	76%	1.00
securitycamera	64%	1.66
slidetray	38%	2.47
smokedetector02	39%	2.38
soundmixer	52%	1.00
speaker04	79%	1.04
spotlight	42%	2.32
studiolight	69%	1.87
taperecorder	100%	0.00
telephone01b	81%	1.12
television	94%	0.39
tripod01	52%	1.79
usbcable01a	94%	0.40
usbkey	94%	0.34
vhstape	64%	1.38
videocamera01a	88%	0.71
videotape01b	73%	1.21
walkietalkie	55%	1.31
walkman	75%	1.16
webcam	82%	1.09
wiicontroller	85%	0.72
wire	58%	1.52
**Food**		
almond *	97%	0.20
apple07 *	97%	0.20
apricot *	100%	0.00
artichoke01b *	97%	0.20
arugula *	100%	0.00
asparagus *	94%	0.41
avocado01 *	100%	0.00
bacon	97%	0.20
bagel01	100%	0.00

bagofpowder	72%	1.62
baguette01	100%	0.00
banana01 *	97%	0.20
beerbottle	70%	1.30
birdfeeder	41%	2.32
birdseed	82%	0.87
blackberry *	97%	0.20
blackolive *	94%	0.39
blueberry01 *	100%	0.00
bluecheese	100%	0.00
bottleofredwine01	97%	0.20
bottleofwhitewine	88%	0.71
bowlofcereal	97%	0.20
bowlofchips	100%	0.00
bread	97%	0.20
breadslice	94%	0.33
broccoli01a *	100%	0.00
brownsugar	82%	1.03
brusselssprout *	97%	0.20
bun	100%	0.00
cabbage *	97%	0.20
candy	97%	0.20
candycane01a	91%	0.52
candydispenser	70%	1.24
cantaloupe01 *	94%	0.39
carrot01 *	97%	0.20
cartonofeggs	91%	0.52
casabamelon *	100%	0.00
cauliflower01 *	97%	0.20
celery *	97%	0.20
champagne	85%	0.72
cherry01 *	97%	0.20
cherrytomato01 *	100%	0.00
chickenshishkebab	100%	0.00
chiliflake	76%	1.17
chocolatechip	91%	0.53
chocolatecroissant	100%	0.00
chocolatemilk	76%	0.80
cocktailshrimp02	94%	0.33
coconut *	91%	0.52
coffeebean *	97%	0.20
cola	85%	0.82
cookie01	100%	0.00
corn02 *	100%	0.00
cracker02	100%	0.00
cremebrulee	94%	0.39
croissant01	100%	0.00

cucumber *	97%	0.20
cupcake	94%	0.39
date *	97%	0.20
donut	100%	0.00
drink	97%	0.20
dumpling	50%	2.17
egg01a	91%	0.52
eggnoodle	100%	0.00
eggplant *	94%	0.39
endive *	100%	0.00
englishcucumber *	100%	0.00
englishmuffin	94%	0.39
fortunecookie	94%	0.39
frenchfries	100%	0.00
fusilli03a	97%	0.20
garlic01a *	97%	0.20
ginger *	94%	0.34
granolabar01	100%	0.00
grape01 *	100%	0.00
greenolive *	100%	0.00
greenonion *	97%	0.20
guacamole	94%	0.39
hamslice	97%	0.20
honeycomb	48%	1.33
honeydewmelon *	100%	0.00
hotdog	100%	0.00
hotdogweiner	88%	0.71
iceberglettuce *	97%	0.20
icecreamcone01a	100%	0.00
icecube01	44%	2.02
icecube02	48%	1.60
jarofapplesauce *	97%	0.20
jarofcapers	94%	0.39
jellybean	97%	0.20
kiwi01a *	100%	0.00
kiwi03 *	100%	0.00
leek *	97%	0.20
lemon02 *	97%	0.20
lettuce *	97%	0.20
lime *	94%	0.39
liquorbottle01	64%	1.24
lollipop01	94%	0.39
lollipop04	94%	0.39
lychee01 *	84%	0.93
macaroni01	100%	0.00
makiroll	100%	0.00
mango01 *	100%	0.00

maplebuttercone	100%	0.00
melbatoast	97%	0.20
milkcontainer	66%	0.93
mushroom01 *	94%	0.39
oats	94%	0.33
onion *	97%	0.20
orange *	100%	0.00
orangejuice	85%	0.72
oyster02	36%	1.97
packofgum	82%	0.91
palmier	91%	0.52
peach01 *	100%	0.00
peanut01 *	94%	0.41
pear01 *	100%	0.00
pecan01 *	88%	0.73
pecan02 *	100%	0.00
penne	100%	0.00
pepper04a *	100%	0.00
pickle01a *	91%	0.60
pineapple01a *	100%	0.00
pintofbeer	84%	0.74
pistachio *	82%	1.01
pitabread	97%	0.20
pizza	97%	0.20
plum01 *	100%	0.00
popcorn	97%	0.20
potato02b *	100%	0.00
pumpkin *	88%	0.65
radish01 *	97%	0.20
raisin *	100%	0.00
raspberry01 *	97%	0.20
rawchicken	94%	0.34
redonion *	94%	0.39
relish	100%	0.00
rice	94%	0.34
salsa	100%	0.00
sand	48%	1.43
sardinecan	97%	0.20
sausage	100%	0.00
shavingcream	61%	1.42
shellpasta	100%	0.00
slushiemachine	52%	1.99
smokedsalmon	94%	0.39
snowpea01 *	91%	0.60
softcheese	100%	0.00
spaghetti01	100%	0.00
springroll	97%	0.20

sprouts *	94%	0.33
strawberry *	91%	0.53
stringbean01 *	100%	0.00
sunflowerseeds *	100%	0.00
swisscheese	88%	0.79
tabascosauce	85%	0.72
teabag	94%	0.39
toast	100%	0.00
tomato01	97%	0.20
tomatojuice	61%	1.87
tortilla	100%	0.00
tortillachip	97%	0.20
turnip *	91%	0.58
walnut01c *	100%	0.00
waterbottle01b	61%	1.69
watermelon01 *	100%	0.00
weddingcake	82%	0.91
zucchini01 *	100%	0.00
**Furniture**		
armchair02	100%	0.00
barrel01	36%	2.88
bed	94%	0.39
bench01	88%	0.73
bookshelf	88%	0.71
boosterseat	70%	1.59
chair	100%	0.00
chest01	58%	1.65
coatrack	70%	1.40
couch02	97%	0.20
desk02	88%	0.78
dresser02	100%	0.00
filingcabinet	61%	1.21
floorlamp	61%	1.54
foldingchair	94%	0.39
footrest02	94%	0.39
gardenswing	88%	0.53
keyhook	33%	2.51
mattress	91%	0.52
mirror02	67%	1.38
nightstand	94%	0.33
officechair01	91%	0.52
officechair03	94%	0.39
outdoorchair	97%	0.20
parkbench02	64%	1.46
patiochair	97%	0.20
picnictable	48%	1.64

pillow01a	36%	1.73
rockingchair	100%	0.00
safe	31%	2.60
shelf	66%	1.67
shelves	81%	1.12
stool01	97%	0.20
table01	100%	0.00
woodencrate01	61%	1.79
**Gamestoy & entertainment**		
8ball	85%	0.61
aceofdiamond	100%	0.00
airhockeytable	85%	0.82
ball02	58%	1.61
beachpaddle01a	79%	0.75
bowlingpin	55%	0.99
bridgestick	52%	1.91
bubbleblower	97%	0.20
chessboard	100%	0.00
chessknight03a	88%	0.78
cigar	36%	2.57
cigarette	32%	2.55
dartboard	88%	0.71
dice05a	94%	0.33
doll	94%	0.39
foosballtable	97%	0.20
gamecontroller01	58%	1.14
gamepiece	91%	0.60
highstrikergame	75%	1.16
horseshoe	41%	1.79
hulahoop	82%	0.80
interlockingbuildingblock03b	94%	0.39
jokercard	100%	0.00
kidpicnictable	55%	1.25
marble	97%	0.20
mrpotatohead	91%	0.58
number3	82%	0.95
pacifier02d	52%	2.14
paddleball	82%	0.68
paperairplane	52%	1.48
pingpongpaddle01a	67%	1.06
pingpongtable	75%	0.95
playground	52%	1.32
playingcard	97%	0.20
pokerchips	100%	0.00
pokerset	97%	0.20
poolcue	76%	1.22

pooltable	85%	0.72
pooltriangle	61%	2.08
puppettheatre	63%	1.30
pusharoundtoy	97%	0.20
puzzle	48%	1.33
puzzlepiece	94%	0.39
rattle	97%	0.20
rockinghorse	88%	0.67
rubikcube	100%	0.00
sandbagthrowgame	67%	1.58
sandshovel02a	64%	1.64
skippingrope	73%	0.98
smokingpipe	33%	2.72
spinningtoy	94%	0.40
stackingring01a	97%	0.20
stickytack	28%	2.69
stuffedanimal02b	88%	0.67
toyanimal01	73%	1.30
toyanimal02	85%	0.82
toyanimal03	85%	0.76
toyanimal04	79%	0.95
toyanimal05	88%	0.65
toydinosaur	67%	1.06
toyfiretruck	52%	1.00
toyshape	82%	0.91
toysoldier01b	76%	1.11
toytank	36%	1.71
toytractor01b	72%	1.00
toyxylophone	82%	0.68
watercolorpaintset	55%	1.52
**Hand labour tool & accessory**		
adjustablewrench01b *	91%	0.52
aircompressor	44%	2.39
axe01 *	94%	0.39
boltcutter *	97%	0.20
bowrake *	76%	1.06
boxcutter03a *	100%	0.00
bucket02	42%	1.68
bungeecord02	48%	1.98
carjack *	59%	1.95
caulking	44%	1.93
cclamp *	97%	0.20
chainsaw *	94%	0.40
cheeseknife01	58%	1.14
chisel02b *	97%	0.20
cuttingpliers02 *	100%	0.00

dolly01 *	81%	1.06
drill01b *	91%	0.58
drillbit03a	75%	1.01
ducttape	56%	1.54
file *	85%	0.90
flashlight02b *	45%	1.85
floodlight	55%	1.64
flooringstapler *	91%	0.52
gardenshears01a *	100%	0.00
gastank	32%	2.11
gavel	31%	2.81
generator	44%	1.81
gluetube	36%	2.63
hammer01 *	97%	0.20
handrake01a *	79%	0.93
hedgeshears *	97%	0.20
hedgetrimmer *	91%	0.53
hoe *	85%	0.61
hosenozzle *	56%	1.49
icescraper *	58%	1.60
jackhammer *	91%	0.58
jigsaw *	73%	1.18
jumpercables	45%	1.49
jumpingjack *	88%	0.71
knifesharpener	58%	1.74
ladder	81%	0.87
lawnmower	58%	1.73
leafblower01 *	78%	0.97
leafrake *	66%	1.27
level03a	79%	1.22
lock03a	36%	2.47
lockingplier *	93%	0.42
loppingshears *	88%	0.78
mallet01b *	88%	0.73
measuringtape01	94%	0.39
metalbrush *	39%	2.05
metalscissors *	81%	0.93
mitresaw *	82%	1.09
nailgun *	94%	0.39
needlenosepliers01 *	97%	0.20
paintbrush01 *	79%	1.14
paintroller01 *	66%	1.57
paintscraper *	75%	1.26
painttray	64%	1.57
pickaxe02 *	91%	0.52
pipewrench *	94%	0.39
pitchfork *	85%	0.82

pliers02b *	94%	0.39
pressurewasher *	66%	1.66
puttyknife *	91%	0.52
pylon01	25%	2.60
rope03	61%	1.81
sandpaper	36%	2.24
saw02b *	91%	0.58
screwdriver04b *	94%	0.39
setsquare *	59%	1.38
sewingmachine01a	44%	1.87
shopvac *	45%	1.43
shovel01 *	88%	0.64
sledgehammer *	97%	0.20
snowblower	70%	1.28
snowbrush *	42%	1.82
snowshovel *	61%	1.59
solderingiron *	53%	1.77
solderingwire	41%	1.97
squeegee01b *	42%	1.53
staplegun *	69%	1.26
stepladder	82%	0.97
stubbywrench01a *	100%	0.00
swissarmyknife01a	44%	2.51
tablesaw *	91%	0.52
tape02a	67%	1.72
toolbox02	79%	0.99
toyhardhat	36%	2.24
wateringcan	39%	1.71
weedwacker *	73%	1.24
wetfloorsign	33%	2.37
wheel01	28%	2.61
wheelbarrow01 *	84%	0.88
wirestripper *	94%	0.39
**Household article & cleaner**		
ashtray01	52%	1.87
ballofstring	30%	2.58
barbecuelighter	27%	2.13
bleachbottle	97%	0.20
broom01	76%	0.93
bucket01a	64%	1.53
carwashmitt	52%	2.12
clothesdryingrack	59%	1.67
clothespin03b	73%	1.53
dishsoap	73%	0.98
doormat	36%	2.20
dryingmachine02	50%	1.90

dustpan	76%	1.14
fireextinguisher01	42%	2.38
flyswatter	47%	2.07
fonduefuel	81%	1.18
garbagebin	56%	1.68
garbagecan02	44%	2.23
glassmop	76%	1.14
handbrush02	58%	1.70
handvacuum	48%	1.65
hanger02a	74%	1.28
iron01b	75%	1.19
ironingboard01	67%	1.54
key01	58%	2.23
key07	58%	2.01
keychain	36%	2.32
laundrybasket01a	85%	0.90
lighter01	45%	2.45
lintbrush	69%	1.36
lintroller	61%	1.69
litterbox	38%	2.43
lunchbox	27%	2.81
match	55%	1.80
motoroilbottle01	52%	2.06
mousetrap	70%	1.48
oblongcan02	55%	1.72
petcarrier	52%	2.28
plantpot	27%	2.43
plasticbasket01a	66%	1.70
plasticgallon	69%	1.47
recyclingbin	66%	1.57
rubberglove02b	39%	1.79
rug01	33%	2.02
safetypin	42%	2.23
scrubbingbrush05b	78%	1.06
shoehorn	35%	2.36
sponge01	73%	0.85
spraybottle01	88%	0.79
spraycan	39%	2.13
suitcase	38%	2.48
thermometer02b	45%	2.31
thimble	45%	2.19
thread02	36%	2.22
vacuumcleaner01	76%	1.11
wallclock	39%	1.69
washingmachine	42%	2.07
windshieldwasherfluid	48%	1.83
yarn	36%	2.37

**Jewel & money**		
50dollarbill	97%	0.20
bracelet01	38%	2.09
coin01b	97%	0.20
crown	36%	1.99
earring01	66%	1.31
necklace	36%	1.87
ring01	73%	1.23
watch02a	36%	1.84
**Kitchen & utensil**		
aluminiumfoil	85%	0.76
bagtie	56%	2.02
bastingbrush	79%	1.14
beermug01a	100%	0.00
blender	94%	0.39
bottlecap	36%	2.32
bottleopener01	88%	0.71
bowl01	94%	0.39
bowl02a	61%	1.56
breadknife	94%	0.33
butterdish	84%	0.78
butterknife	79%	1.05
cakeplatter	82%	1.03
canister	75%	1.01
chocolatefonduedish	48%	1.75
cleaver01	69%	1.04
cleaver02	91%	0.60
coffeemaker	91%	0.52
coffeepot03a	97%	0.20
contactlens	66%	1.42
cookiecutter	88%	0.64
cookingpot	100%	0.00
cork02	61%	1.92
corkscrew03a	70%	1.16
cowbell	19%	3.18
cutleryset	91%	0.52
cuttingboard	78%	1.10
dishrack	56%	2.03
drinkshaker	91%	0.60
eggslicer	85%	0.90
electricmixer	91%	0.44
espressomachine	73%	1.35
flask	53%	2.26
fonduefork01	85%	0.82
foodprocessor	73%	1.24

fork03c	97%	0.20
fork07b	94%	0.39
freezer02	39%	2.27
fridge	64%	1.30
fryingpan02a	100%	0.00
funnel	70%	1.53
garlicpress02a	97%	0.20
gasburner	78%	1.16
glass02a	36%	2.13
grater01a	94%	0.39
gravyboat	75%	1.08
grill	79%	1.14
handblender	91%	0.52
handmixer01d	88%	0.63
hourglass	30%	2.51
huntingknife	50%	1.90
icebucket	72%	1.26
icecubetray01a	85%	0.90
icepack	70%	1.54
jar02	58%	1.70
jar03	88%	0.71
jug	85%	0.76
kettle01	97%	0.20
kitchenscale01a	73%	0.98
knife03	94%	0.39
ladle02a	97%	0.20
martiniglass01b	97%	0.20
measuringcup01	97%	0.20
measuringspoon	97%	0.20
microwave	55%	1.32
minifridge	45%	1.81
mortarandpestle	82%	1.09
muffintray01	97%	0.20
muffintray02	100%	0.00
mug01	97%	0.20
mug05	88%	0.71
musicalwoodenspoons	59%	1.29
nutcracker01	67%	1.48
oven	67%	1.35
paninigrill	76%	1.28
pastaspoon	97%	0.20
peeler01	91%	0.58
peppermill02b	85%	0.61
pitcher02b	91%	0.52
plate01b	100%	0.00
pot01	45%	1.36
pot02a	97%	0.20

potatomasher03b	100%	0.00
ramekin01	94%	0.39
rollingpin01a	97%	0.20
saladspinner	84%	0.74
saltshaker03a	64%	1.10
servingspoon01	100%	0.00
shotglass	88%	0.73
sieve01b	97%	0.20
sippycup	73%	1.44
spatula03	100%	0.00
spatula04	97%	0.20
spicerack	79%	0.87
spoon01	97%	0.20
stovetop	91%	0.52
strainer02	97%	0.20
straw	78%	1.31
styrofoamcup	85%	0.97
tableknife02	94%	0.40
teapot	85%	0.72
thermos	42%	2.69
toaster01	82%	0.80
toasteroven	76%	0.80
tongs01b	100%	0.00
toothpick02	48%	2.64
towel01	58%	1.80
travelmug	88%	0.71
tray	67%	1.51
tupperware03a	91%	0.58
wafflemaker	88%	0.53
waterbottle03	48%	1.59
watercooler	36%	2.40
whisk	97%	0.20
wineglass01	100%	0.00
winerack	48%	1.99
woodenspoon	94%	0.39
**Medical instrument & accessory**		
bandage	85%	0.82
bandaid01	53%	1.48
cane	58%	1.83
dropper01	76%	1.11
eyechart	72%	1.37
eyedrops	44%	2.42
microscope	63%	1.41
nasalspray01	36%	2.30
pill	82%	1.03
pillbottle	82%	0.91

scalpel	56%	1.69
syringe01	91%	0.44
syringe02	100%	0.00
ziplocbag	38%	2.45
**Musical instrument**		
accordion01 *	100%	0.00
acousticguitar02 *	100%	0.00
balalaika *	97%	0.20
banjo *	100%	0.00
bassguitar *	100%	0.00
cabasa *	73%	1.32
cello *	100%	0.00
chime *	94%	0.33
clarinet *	97%	0.20
congadrum *	97%	0.20
cornet *	94%	0.39
cymbal *	94%	0.40
doublebass *	100%	0.00
drum01 *	97%	0.20
drumset *	97%	0.20
drumstick *	91%	0.58
electricdrumset *	88%	0.71
electricguitar01 *	100%	0.00
flugglehorn *	94%	0.33
frenchhorn *	97%	0.20
gong *	85%	0.90
grandpiano *	100%	0.00
guitarcase	91%	0.58
harmonica *	94%	0.39
hihat *	100%	0.00
keyboard *	88%	0.65
maraca01 *	76%	1.32
metronome	45%	2.19
musicsheet	79%	1.04
musicstand	81%	1.06
rainstick *	52%	2.01
record	52%	1.62
recorder01 *	94%	0.39
recorder04 *	91%	0.52
saxophone *	100%	0.00
shoepolish	48%	2.31
tambourine01 *	85%	0.72
tambourine03 *	97%	0.20
triangle *	91%	0.58
trombone *	94%	0.40
trumpet *	97%	0.20

tuba *	97%	0.20
uprightpiano01 *	100%	0.00
violin *	100%	0.00
xylophone *	100%	0.00
**Natural element**		
acorn	48%	1.17
aloe01	67%	1.34
beehive	82%	1.09
birdnest	76%	1.22
branch02	88%	0.78
cactus	64%	1.46
campfire	64%	1.52
cattail	94%	0.34
cloud	97%	0.20
clover	94%	0.39
craneflower	94%	0.33
daffodil	97%	0.20
daisy	97%	0.20
dandelion	97%	0.20
feather03a	45%	1.79
fern	94%	0.40
firtree	70%	1.16
flower01	97%	0.20
goldnugget	69%	1.51
hibiscusflower02	94%	0.39
inukshuk	52%	1.64
leaf02a	100%	0.00
lily	91%	0.52
lilypad	100%	0.00
log	82%	1.09
moon	67%	1.54
pinecone01	79%	0.87
plant01	63%	1.49
plasticflower01a	82%	0.68
rock01a	82%	1.01
rose	97%	0.20
soil	67%	1.42
spiderweb	61%	1.70
tree	97%	0.20
treestump	100%	0.00
treetrunk	91%	0.52
tulip02	91%	0.52
waterfall	91%	0.53
waterlily	97%	0.20
wheat	58%	0.98

Outdoor activity & sport item		
anchor	34%	2.69
backfloat	44%	1.62
backpack01a	61%	1.60
badmintonracket	94%	0.33
barbecue03	45%	2.12
baseball01a	73%	0.85
baseballbat	88%	0.63
baseballglove	85%	0.76
basketball01	94%	0.33
basketballhoop01	91%	0.52
beachumbrella01	67%	1.46
bicycle	50%	1.00
bikehelmet	85%	0.72
bikelock	58%	1.88
bikepump01	63%	1.45
bikewheel	61%	1.12
binoculars01b	70%	1.51
birdie	76%	0.80
boatmotor	45%	1.84
boogieboard	73%	1.18
bowlingball	70%	0.88
boxingglove01	85%	0.84
canoepaddle02	91%	0.58
catchermask	85%	0.82
charcoalbarbecue	38%	2.31
cooler01	52%	1.72
crosscountryski	94%	0.39
diaperbag	35%	2.53
downhillski	97%	0.20
ellipticalmachine	82%	0.91
exercisebench	73%	1.18
exercisebike	73%	1.32
exercisemachine	82%	0.95
fishinghook03	90%	0.56
fishingrod	97%	0.20
football	94%	0.33
footballhelmet	85%	0.72
frisbee	67%	1.06
golfbag	91%	0.44
golfball	91%	0.52
gymnasticring	71%	1.29
handgripper	67%	1.42
hockeygoalieglove	91%	0.58
hockeygoaliemask	91%	0.44
hockeygoaliepad	78%	1.10
hockeypuck	85%	0.82

hockeystick	94%	0.33
hose	45%	1.72
iceskate	85%	0.72
kiddiepool	78%	1.01
kite	52%	1.00
kneepad01c	82%	1.03
lifejacket	73%	1.11
lifesaver	72%	1.44
magneticcompass	42%	2.57
outdoorheater	32%	2.49
parachute	73%	1.21
patioumbrella	42%	1.85
podium	39%	2.28
poolnet	66%	1.69
propanetank01	47%	2.17
punchingbag	85%	0.61
punchingball	91%	0.52
racquetballracket	81%	0.93
reflector	42%	2.25
rollerblade	91%	0.52
shinpad	88%	0.53
shoulderpad	76%	1.32
skateboard	71%	1.14
skiboot01a	91%	0.52
skigoggles01	85%	0.61
skihelmet01	79%	0.99
skipole	100%	0.00
snowboard	97%	0.20
snowman	41%	1.98
snowshoe	94%	0.39
soccerball	88%	0.53
speedball	97%	0.20
sportbag	48%	2.16
stroller	39%	2.05
surfboard	88%	0.73
swimgoggles	88%	0.64
swing	41%	1.84
telescope	24%	2.75
tennisball01b	85%	0.72
tennisracket	97%	0.20
tent	66%	1.23
toyhockeystick01	52%	1.00
trampoline	64%	1.32
treadmill	67%	1.54
umbrella04	39%	2.19
volleyball	88%	0.53
waterbottleholder	47%	2.41

weight01	78%	1.22
**Skincare & bathroom item**		
bathtub	53%	1.73
comb02a	94%	0.39
compactpowder	88%	0.71
condom	30%	2.73
cottonpad	88%	0.78
curlingiron01a	64%	1.59
dentalfloss03b	91%	0.58
deodorant02a	94%	0.39
electricrazor	82%	0.80
electrictoothbrush01c	94%	0.33
faucet	48%	2.04
hairclip02	48%	2.16
hairclip03	58%	1.59
hairclipper	61%	1.48
hairdryer02a	66%	1.16
hairpin	69%	1.45
lipstick02a	94%	0.39
makeupbrush04	94%	0.39
mascarabrush	91%	0.53
nailclipper03b	88%	0.71
nailfile	78%	1.01
nailpolish03b	91%	0.58
papertowel	36%	1.84
perfume01a	94%	0.40
plunger02	52%	1.48
qtip	94%	0.39
razor01	97%	0.20
scale01a	58%	1.80
shower	48%	2.04
showerhead01	58%	1.76
sink	63%	1.65
soap03a	70%	1.51
soapdispenser01	94%	0.33
tampon	81%	0.93
tissuebox01b	59%	1.44
toilet	75%	1.29
toiletbrush	48%	1.33
toiletpaper01a	97%	0.20
toothbrush03b	94%	0.39
toothpastetube	88%	0.63
towel04a	69%	1.42
towelrack	58%	1.90
tube	73%	1.53
tweezers02a	88%	0.63

urinal	70%	1.19
**Stationary & school supply**		
bag	33%	2.56
binder03b	97%	0.20
book01b	67%	1.60
calculator01	56%	0.99
calendar	88%	0.63
callbell	30%	2.97
cardboardbox	50%	1.86
chalk	78%	1.16
chalkboard01	75%	1.25
chalkboarderaser01	91%	0.52
clipboard	100%	0.00
coloringset	45%	1.82
corkboard	73%	1.51
crayon	61%	1.12
crumpledpaper	58%	2.09
diploma01	45%	1.51
diploma02	67%	1.21
divider02	94%	0.39
envelope03a	94%	0.40
eraser	97%	0.20
expandablefolder03a	100%	0.00
firstaidkit	27%	2.91
folder	100%	0.00
fountainpen	100%	0.00
globe	38%	2.19
gluestick	82%	1.09
highlighter02b	85%	0.84
linedpaper	100%	0.00
lunchbag	36%	2.47
magnifyingglass01b	36%	2.41
marker01a	88%	0.63
mathcompass	73%	1.12
mechanicalpencil02	100%	0.00
nametag01a	50%	2.32
notebook03a	100%	0.00
oneholepunch	61%	1.27
paperclip01b	97%	0.20
paperclip03	84%	0.86
papertray	72%	1.39
pen04b	97%	0.20
pencil01	94%	0.39
pencilcase01	75%	1.44
pencilsharpener02a	91%	0.60
postitnote	42%	1.82

projectorscreen	50%	2.11
protractor	88%	0.71
rubberband01	81%	0.97
ruler04	91%	0.52
scissors01	56%	1.70
scotchtape	94%	0.39
smartboard	72%	1.13
staple	88%	0.79
stapler03a	94%	0.39
stapleremover	88%	0.71
storagebin	41%	2.13
threeholepunch03	91%	0.52
thumbtack02a	85%	0.82
triangleruler01	94%	0.40
whiteout	91%	0.53
worldmap	67%	1.15
**Vehicle**		
battleship *	74%	1.03
boat *	79%	1.10
boxtrailer *	88%	0.71
boxtruck *	97%	0.20
bulldozer *	91%	0.53
bumpercar	61%	1.27
bus *	100%	0.00
car *	97%	0.20
carkey	47%	2.14
carmat	36%	2.47
carsidemirror01	67%	1.58
cementtruck *	94%	0.39
cessna *	97%	0.20
cherrypicker *	82%	0.80
convertible *	97%	0.20
cruiseship *	88%	0.78
dogsled	58%	1.73
dumptruck *	100%	0.00
ferry *	97%	0.20
fighterjet *	76%	1.11
forklift *	87%	0.75
freighttruck *	97%	0.20
gardenutilityvehicle *	100%	0.00
gearshift	61%	1.69
headlight	58%	1.75
helicopter *	82%	0.85
hubcap	67%	1.54
jeep *	100%	0.00
jetski *	85%	0.61

kidbicycle *	50%	1.51
lawnmowertractor *	94%	0.35
licenseplate	55%	2.04
locomotive *	97%	0.20
mailtruck *	94%	0.39
motorboat02 *	87%	0.55
motorcycle *	100%	0.00
porsche *	100%	0.00
powerchair	91%	0.44
rearviewmirror	61%	1.99
rowboat *	77%	0.77
sailboat *	94%	0.39
schoolbus *	94%	0.40
scissorlift *	79%	0.95
scooter *	44%	1.51
shoppingcart	41%	2.31
spacerocket	67%	1.48
spacerover	70%	1.42
steamroller *	97%	0.20
steeringwheel	67%	1.42
taillight	45%	2.06
tank *	64%	0.95
tankertruck *	100%	0.00
taxisign	55%	2.05
tire	63%	1.55
toycar04	79%	0.75
toytanktruck	59%	0.97
tractor *	97%	0.20
trailer *	88%	0.78
tricycle *	58%	1.40
wagonwheel	42%	1.94
wheel04	45%	2.07
wheelchair	58%	1.45
windshieldwiper02	48%	2.10
windsurfboard	52%	1.32
**War related weapon & item**		
arrow02	76%	0.80
battleaxe	97%	0.20
bazooka	85%	0.84
bow	58%	1.53
brassknuckle	55%	2.24
broadsword	94%	0.39
bullet	91%	0.58
bulletbelt	97%	0.20
cannon	94%	0.39
centurionhelmet	72%	1.26

chainmail	70%	1.03
cutlass	84%	0.78
flail	97%	0.20
flintlockpistol	94%	0.39
halberd	88%	0.78
handcuffs	70%	1.63
handheldtelescope	33%	2.43
harpoon	81%	0.99
helmet	82%	0.91
joustingspear	47%	2.18
kalashnikov	97%	0.20
machete	50%	1.66
machinegun	97%	0.20
missile	70%	1.36
morningstar	67%	1.65
musket	97%	0.20
nunchuk	61%	1.56
pirateflag	45%	1.51
revolver	88%	0.78
shield02	78%	0.95
spear02	85%	0.97
suitofarmor	73%	0.98
sword01	91%	0.60
tonfa	52%	2.16
trident01	66%	1.74
vikingmask01b	72%	1.35

CA = Category Agreement

* denotes a stimulus that was included in the analyses of categories.
